# Monoamine oxidase inhibition properties of 2,1-benzisoxazole derivatives

**DOI:** 10.1007/s11030-023-10628-4

**Published:** 2023-03-19

**Authors:** Anton Shetnev, Alexandr Kotov, Anna Kunichkina, Irina Proskurina, Sergey Baykov, Mikhail Korsakov, Anél Petzer, Jacobus P. Petzer

**Affiliations:** 1 Pharmaceutical Technology Transfer Center, Yaroslavl State Pedagogical University Named After K.D. Ushinsky, 108 Respublikanskaya St., Yaroslavl, 150000 Russian Federation; 2 Department of Organic Chemistry, Kosygin Russian State University, 115035 Moscow, Russia; 3 Institute of ChemistryDepartment of Organic Chemistry, Kosygin Russian State University, 115035, Moscow, Russia, Saint Petersburg State University, 7/9 Universitetskaya Nab., Saint Petersburg, 199034 Russian Federation; 4 Pharmaceutical Chemistry and Centre of Excellence for Pharmaceutical Sciences, North-West University, Potchefstroom, 2520 South Africa

**Keywords:** Monoamine oxidase, MAO, Inhibition, 2,1-benzisoxazole, Specificity

## Abstract

Monoamine oxidase (MAO) are flavoenzymes that metabolize neurotransmitter, dietary and xenobiotic amines to their corresponding aldehydes with the production of hydrogen peroxide. Two isoforms, MAO-A and MAO-B, are expressed in humans and mammals, and display different substrate and inhibitor specificities as well as different physiological roles. MAO inhibitors are of much therapeutic value and are used for the treatment of neuropsychiatric and neurodegenerative disorders such as depression, anxiety disorders, and Parkinson’s disease. To discover MAO inhibitors with good potencies and interesting isoform specificities, the present study synthesized a series of 2,1-benzisoxazole (anthranil) derivatives and evaluated them as in vitro inhibitors of human MAO. The compounds were in most instances specific inhibitors of MAO-B with the most potent MAO-B inhibition observed for **7a** (IC_50_ = 0.017 µM) and **7b** (IC_50_ = 0.098 µM). The most potent MAO-A inhibition was observed for **3l** (IC_50_ = 5.35 µM) and **5** (IC_50_ = 3.29 µM). It is interesting to note that 3-(2-aminoethoxy)-1,2-benzisoxazole derivatives, the 1,2-benzisoxazole, zonisamide, as well as the isoxazole compound, leflunomide, have been described as MAO inhibitors. This is however the first report of MAO inhibition by derivatives of the 2,1-benzisoxazole structural isomer.

**Graphical abstract:**

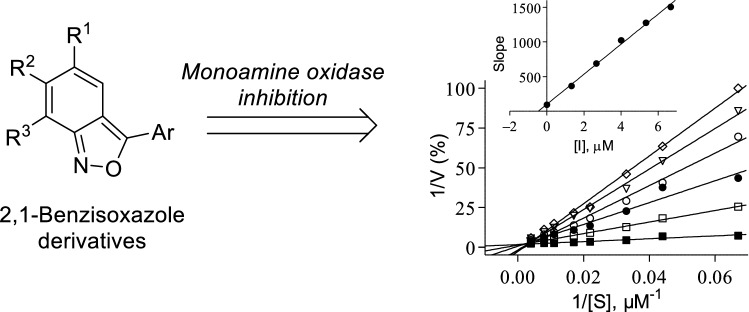

**Supplementary Information:**

The online version contains supplementary material available at 10.1007/s11030-023-10628-4.

## Introduction

The monoamine oxidase (MAO) enzymes are flavoenzymes that are bound to the outer membranes of mitochondria [1]. MAO catalyzes the α-carbon oxidation of neurotransmitter, dietary, and xenobiotic amines to yield the corresponding aldehydes, ammonia (for primary amine substrates) and hydrogen peroxide [2]. Two isoforms, MAO-A and MAO-B, are expressed in humans and mammals and are products of distinct genes [3, 4]. The two isoforms exhibit different tissue distributions, substrate and inhibitor specificities and physiological roles. For example, serotonin is a specific substrate for MAO-A, while benzylamine and phenethylamine are MAO-B specific substrates [5]. Certain amine compounds such as dopamine, epinephrine, norepinephrine, and tyramine are substrates for both MAO isoforms. Since MAO metabolizes neurotransmitters and other biogenic amines, they are of physiological and therapeutic importance. In this respect, MAO-A inhibitors have been used for decades for the treatment of depression and anxiety disorders while MAO-B inhibitors are used for Parkinson’s disease therapy, often in combination with levodopa, the metabolic precursor of dopamine [5]. In Parkinson’s disease, MAO-B inhibitors provide symptomatic benefit by reducing the MAO-mediated metabolism of dopamine in the brain [6, 7]. The use of MAO-A inhibitors in depression is based on the monoamine hypothesis of depression, and an antidepressant effect is obtained by enhancing central serotonin and norepinephrine levels [8–10].

Although MAO inhibitors act by increasing the biological half-lives of monoamine neurotransmitters, they also reduce the formation of the metabolic by-products of the MAO catalytic cycle. Hydrogen peroxide that is generated by the reduction of molecular oxygen during MAO catalysis may be converted to injurious oxygen species in the brain, and may contribute to neurodegeneration in Parkinson’s disease [2]. MAO-B inhibitors have thus been advocated as potential neuroprotective agents that may delay neurodegeneration by reducing oxidative damage to susceptible neuronal tissues. The production of hydrogen peroxide by MAO-A in the heart and resultant oxidative damage to mitochondria, in turn, have been linked to heart failure [11, 12]. MAO-A inhibitors may thus have a future role in the treatment of cardiovascular disease. Finally, growth and metastasis of prostate cancer is reduced by MAO inhibitors in pre-clinical models while clinical evidence has demonstrated that MAO inhibitors (e.g., phenelzine) may represent a new treatment for prostate cancer [13, 14].

**Fig. 1:**
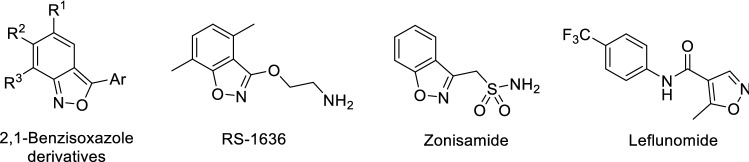
The general structure of the 2,1-benzisoxazole derivatives that were investigated in this study as well as the structures of 3-(2-aminoethoxy)-1,2-benzisoxazole derivative RS-1636, zonisamide and leflunomide

Based on their current and future clinical applications as well as an academic interest in the discovery of new MAO inhibitors [15], the present study synthesized a series of 2,1-benzisoxazole (anthranil) derivatives and evaluated them as in vitro inhibitors of human MAO (Fig. 1). This is the first report of MAO inhibition by 2,1-benzisoxazoles, however, derivatives of the structural isomer, 1,2-benzisoxazole, have been reported to inhibit MAO. For example, 3-(2-aminoethoxy)-1,2-benzisoxazole derivatives (e.g., RS-1636), the 1,2-benzisoxazole, zonisamide, as well as the isoxazole compound, leflunomide, have been described as MAO inhibitors [16–18]. Zonisamide is of particular interest since the X-ray crystal structure of this compound in complex with human MAO-B has been reported [19]. Zonisamide is a competitive inhibitor of human MAO-B (K_i_ = 3.1 μM) and binds reversibly to the substrate cavity of the enzyme. Interestingly, like safinamide bound to MAO-B, zonisamide binds to MAO-B with the Ile-199 gating residue in the “open” conformation [20]. In contrast, when the small molecule inhibitor, isatin, binds to MAO-B, the Ile-199 residue adopts the “closed” conformation [21]. This study synthesized a series of 2,1-benzisoxazole derivatives with divergent structures with respect to the substituents and substitution patterns. The selection of the derivatives was primarily based on synthetic feasibility and derivatives were obtained by a method developed by us earlier. While the 2,1-benzisoxazole derivatives were not designed with a specific medicinal chemistry rationale in mind, this is an exploratory study to evaluate the possibility that the 2,1-benzisoxazole moiety could be used as scaffold for the future design of MAO inhibitors.

## Results and discussion

### Chemistry

**Fig. 2:**
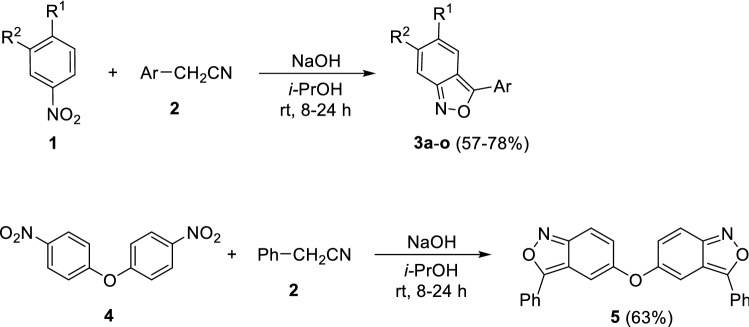
The synthesis of 2,1-benzisoxazole derivatives **3a**–**o** and the bis-condensation product **5**

A variety of clinically used drugs are benzisoxazole derivatives (e.g., leflunomide, oxacillin, dicloxacillin, danazol, risperidone, and zonisamide). Several methods are known for the synthesis of 2,1-benzisoxazoles from ortho-substituted benzene derivatives that contain substituents suitable for cyclization to yield the fused isoxazole ring [22, 23]. In this work, an experimental series of 2,1-benzisoxazoles was obtained by our earlier developed method of condensation of nitroaromatic substrates with arylacetonitriles by heating in an alcoholic alkaline medium (Fig. 2) [24]. This transformation proceeds smoothly only if there is a substituent on the *para-*position of nitroaromatic substrate **1**. In addition, the formation of 2,1-benzisoxazoles is not observed for nitro compounds containing donor groups on the *para*-position, such as primary, secondary or tertiary amino groups, methyl or hydroxyl groups. In addition, the substituent should not be an active nucleofuge under the reaction conditions or easily enters into nucleophilic addition reactions. Despite these limitations, this reaction was used to synthesize a diverse series of 2,1-benzisoxazole derivatives **3a**–**o** that are suitable for probing the structure–activity relationships of this class. The bis-condensation product **5** was obtained in a similar manner.

**Fig. 3:**
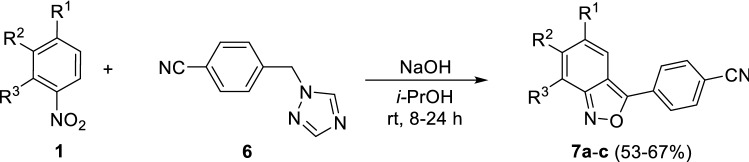
The synthesis of 2,1-benzisoxazole derivatives **7a**–**c**

**Fig. 4:**
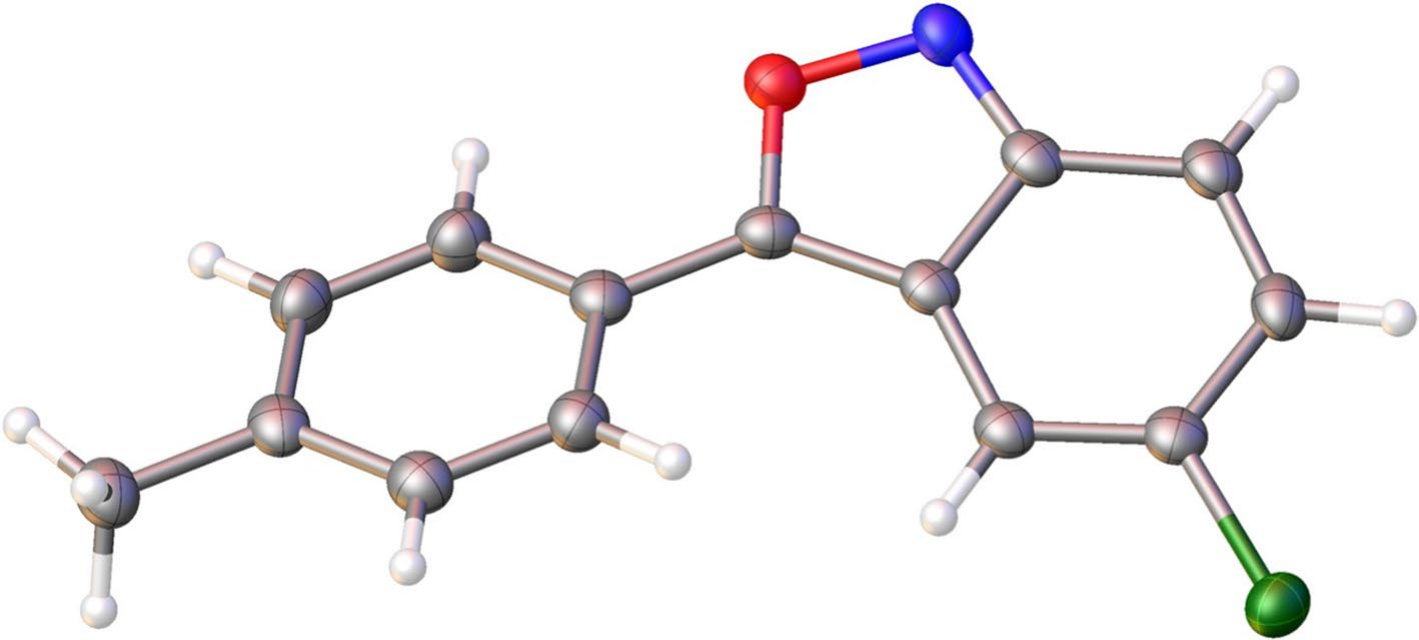
OLEX2 view of **3c** displaying thermal ellipsoids at 50% probably level

For the synthesis of **7a**–**c**, a new approach was used with 4-cyanophenylmethyl-substituted triazole as reagent instead of an arylacetonitrile (Fig. 3). It is postulated that during the reaction, the 1,2,4-triazole fragment stabilizes the carbanion of the methylene group of reagent **6** due to its electron-withdrawing properties, and during the second stage it acts as a leaving group. As a result, the ring closes to form the benzisoxazole compounds **7a**–**c**. We believe that this synthetic protocol is the more accessible and safe modification of that reported in Fig. 2 due to the absence of cyanide-containing waste. Also, Reagent **6** is a commercially available intermediate used in the synthesis of the active pharmaceutical ingredient letrozole [25]. Similar *N*-alkylated triazoles are readily available by reaction between sodium triazolate and the corresponding benzyl halide [26]. It should be noted that for this transformation, the same restrictions on the structure of the nitroaromatic substrates apply as described for Fig. 2. The structures of all the synthesized compounds were confirmed by NMR spectroscopy and high-resolution mass spectrometry. Moreover, the structure of **3c** was established by X-ray diffraction analysis (Fig. 4).

### MAO inhibition studies

**Fig. 5:**
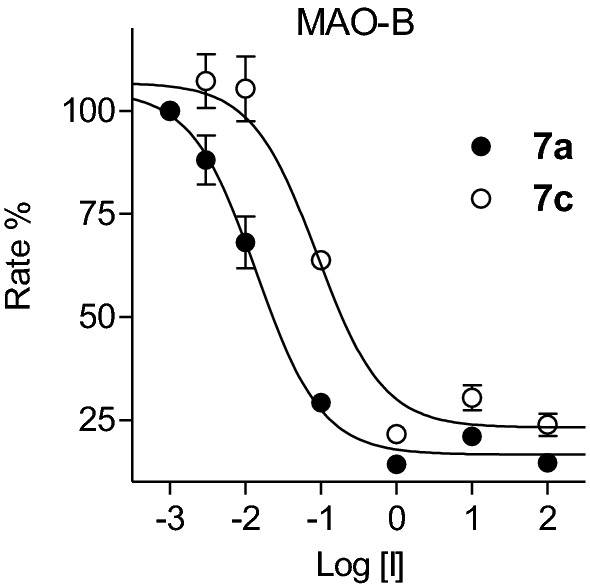
Plots of MAO-B catalytic rate versus inhibitor concentration (log[I]) for **7a** (filled circles) and **7c** (open circles)

The MAO inhibition properties of the 2,1-benzisoxazole derivatives were investigated using the commercially available recombinant human MAO-A and MAO-B enzymes with kynuramine serving as substrate [27, 28]. MAO oxidizes kynuramine to yield 4-hydroxyquinoline, which was measured by fluorescence spectrophotometry [27, 28]. By measuring MAO catalytic rate in the presence of a range of inhibitor concentrations (0.003–100 µM), sigmoidal plots of rate versus the logarithm of inhibitor concentration were constructed from which IC_50_ values were estimated (Fig. 5). The IC_50_ values are presented in Table 1 and show that the 2,1-benzisoxazole derivatives are indeed MAO inhibitors.

**Table 1:** The human MAO inhibition potencies of 2,1-benzisoxazole derivatives **3a**–**o, 5, 7a**–**c** and reference compounds

		IC_50_ (µM ± SD)^a^		
		MAO-A	MAO-B	SI^b^
**3a**		19.4 ± 2.58	3.03 ± 0.184	6.4
**3b**		77.7 ± 5.97	6.59 ± 0.111	12
**3c**		8.76 ± 0.058	2.63 ± 0.178	3.3
**3d**		71.6 ± 3.08	7.92 ± 0.684	9.0
**3e**		8.95 ± 0.581	3.55 ± 0.037	2.5
**3f**		NI^c^	25.1 ± 2.412	–
**3g**		19.5 ± 3.20	5.79 ± 0.013	3.4
**3h**		17.7 ± 0.424	16.3 ± 0.113	1.1
**3i**		43.9 ± 11.3	3.49 ± 0.056	13
**3j**		10.6 ± 0.049	4.43 ± 0.120	2.4
**3k**		NI^c^	5.56 ± 0.730	–
**3l**		5.35 ± 0.211	27.3 ± 2.16	0.20
**3m**		83.2 ± 33.3	1.18 ± 0.022	71
**3n**		81.3 ± 28.7	2.07 ± 0.381	39
**3o**		8.49 ± 0.017	2.34 ± 0.055	3.6
**5**		3.29 ± 0.254	10.0 ± 0.949	0.33
**7a**		19.0 ± 1.20	0.017 ± 0.0045	1138
**7b**		61.0 ± 9.19	0.098 ± 0.0036	622
**7c**		NI^c^	0.139 ± 0.044	–
Harmine^d^		0.0041 ± 0.00007	NI^c^	–
Isatin^d^		12.3 ± 1.74	4.86 ± 0.707	2.5

^a^Values are given as the mean ± standard deviation (SD) of triplicate determinations

^b^Selectivity index: SI = IC_50_(MAO-A)/IC_50_(MAO-B)

^c^No inhibition observed at 100 µM

^d^Reference MAO inhibitor

From the MAO-B inhibition data, the following observations and structure–activity relationships are apparent: (a) The most potent MAO-B inhibition was observed for **7a** (IC_50_ = 0.017 µM) and **7b** (IC_50_ = 0.098 µM) while the most potent MAO-A inhibition was observed for **5** (IC_50_ = 3.29 µM) and **3l** (IC_50_ = 5.35 µM); (b) With the exception, of **5** and **3l**, all compounds displayed specificity for the MAO-B isoform. Compounds **7a** and **7b** may be highlighted as displaying high specificity and potency for MAO-B; (c) The most potent MAO-B inhibition was observed for the *p*-benzonitrile substituted compounds **7a**–**c**, which demonstrates that this group is more optimal than the other aryl groups considered. Thus, **7b** was at least 12-fold more potent than the corresponding *p*-tolyl (**3c**), phenyl (**3j**), *p*-Br-phenyl (**3m**), *p*-Cl-phenyl (**3n**) and *p*-OCH_3_-phenyl (**3o**) homologues. Similarly, **7c** was more potent than the phenyl homologue **3e**; (d) Among the iodo derivatives, the *p*-OCH_3_-phenyl (**3a**), *p*-Cl-phenyl (**3b**) and tolyl (**3i**) substituted compounds were the most potent MAO-B inhibitors with IC_50_ < 6.59 µM. Weaker MAO-B inhibition was observed with the phenyl (**3h**; IC_50_ < 16.3 µM) which demonstrates the requirement of a substituent on the phenyl; (e) Among the halogens on C5, chloro substitution (**3j**) led to slightly more potent MAO-B inhibition than bromo substitution (**3g**), while iodo substitution (**3h**) resulted in comparatively weak inhibition. Larger groups such as 1,3-dioxolanyl (e.g., **3l**) on C5 also yielded lower potency MAO-B inhibitors; (f) Interestingly, the two compounds substituted with the large 5-methyl-1,2,4-oxadiazolyl group (**3e** and **7c**) displayed good MAO-B inhibition potency. In fact, **7c** was the third most potent MAO-B inhibitor of the series. This finding shows that a large group on C6 is well tolerated for MAO-B inhibition.

From the MAO-A inhibition data, the following observations and structure–activity relationships are apparent: (a) The most potent MAO-A inhibition was observed for **5** (IC_50_ = 3.29 µM) and **3l** (IC_50_ = 5.35 µM). Compound **5** is the bis-condensation product; (b) As mentioned above, only **5** and **3l** displayed specificity for the MAO-A isoform, although only by a small degree; (c) Among the C5 chloro substituted compounds the *p*-tolyl (**3c**) and phenyl (**3j**) substituted compounds were more optimal than the other aryl groups considered [e.g., *p*-Br-phenyl (**3m**), *p*-Cl-phenyl (**3n**) and *p*-benzonitrile (**7b**)]; (d) The C5 iodo and bromo derivatives proved to be relatively weak MAO-A inhibitors with IC_50_ > 17.7 µM; (e) Among the halogens on C5, chloro substitution (**3j**) led to more potent MAO-A inhibition than iodo (**3h**) and bromo substitution (**3g**); (f) Interestingly, the 1,3-dioxolanyl substituted compound (**3l**) yielded good MAO-A inhibition (IC_50_ = 5.35 µM).

### Competitive mode of MAO inhibition

**Fig. 6:**
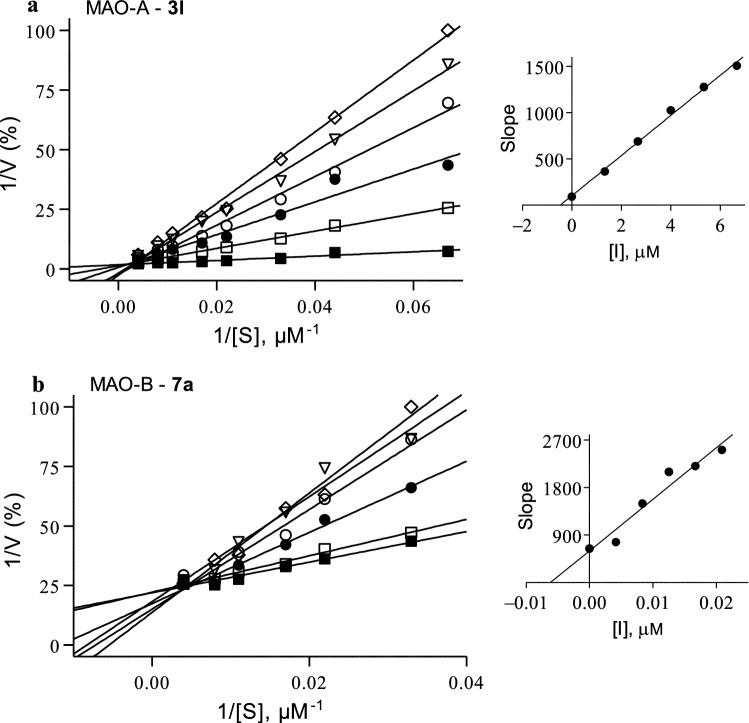
Lineweaver–Burk plots for the inhibition of MAO-A and MAO-B by **3l** (**a**) and **7a** (**b**), respectively. The following inhibitor concentrations were used: 0 × IC_50_ (filled squares), ¼ × IC_50_ (open squares), ½ × IC_50_ (filled circles), ¾ × IC_50_ (open circles), 1 × IC_50_ (triangles) and 1¼ × IC_50_ (diamonds). The insets are replots of the slopes of the Lineweaver–Burk plots versus inhibitor concentration. The K_i_ value equals the negative of the x-axis intercept when y = 0

To obtain insight into the modes of inhibition of the 2,1-benzisoxazoles, Lineweaver–Burk plots for the inhibition of MAO-A and MAO-B by **3l** and **7a**, respectively, were constructed. These two compounds represent potent MAO inhibitors among the study compounds. For each compound, a set of Lineweaver–Burk plots were constructed at the following inhibitor concentrations: 0 × IC_50_, ¼ × IC_50_, ½ × IC_50_, ¾ × IC_50_, 1 × IC_50_ and 1¼ × IC_50_. Each line of the Lineweaver–Burk plots was constructed with the kynuramine concentration ranging from 15 to 250 μM. The Lineweaver–Burk plots for the inhibition of MAO by **3l** and **7a** are presented in Fig. 6 and show that in both instances the lines intersect on the y-axis which is indicative of competitive and therefore reversible inhibition. From a replot of the slopes of the Lineweaver–Burk plots versus inhibitor concentration, a K_i_ value of 0.46 µM is estimated for the inhibition of MAO-A by **3l** (K_i_ = −x when y = 0). For the inhibition of MAO-B by **7a**, a K_i_ value of 0.0062 µM is estimated. The K_i_ values have also been determined by global (shared) fitting of the inhibition data to the Michaelis–Menten equation: 0.50 ± 0.024 µM (**3l**, MAO-A) and 0.010 ± 0.0015 µM (**7a**, MAO-B).

### Molecular docking

**Fig. 7:**
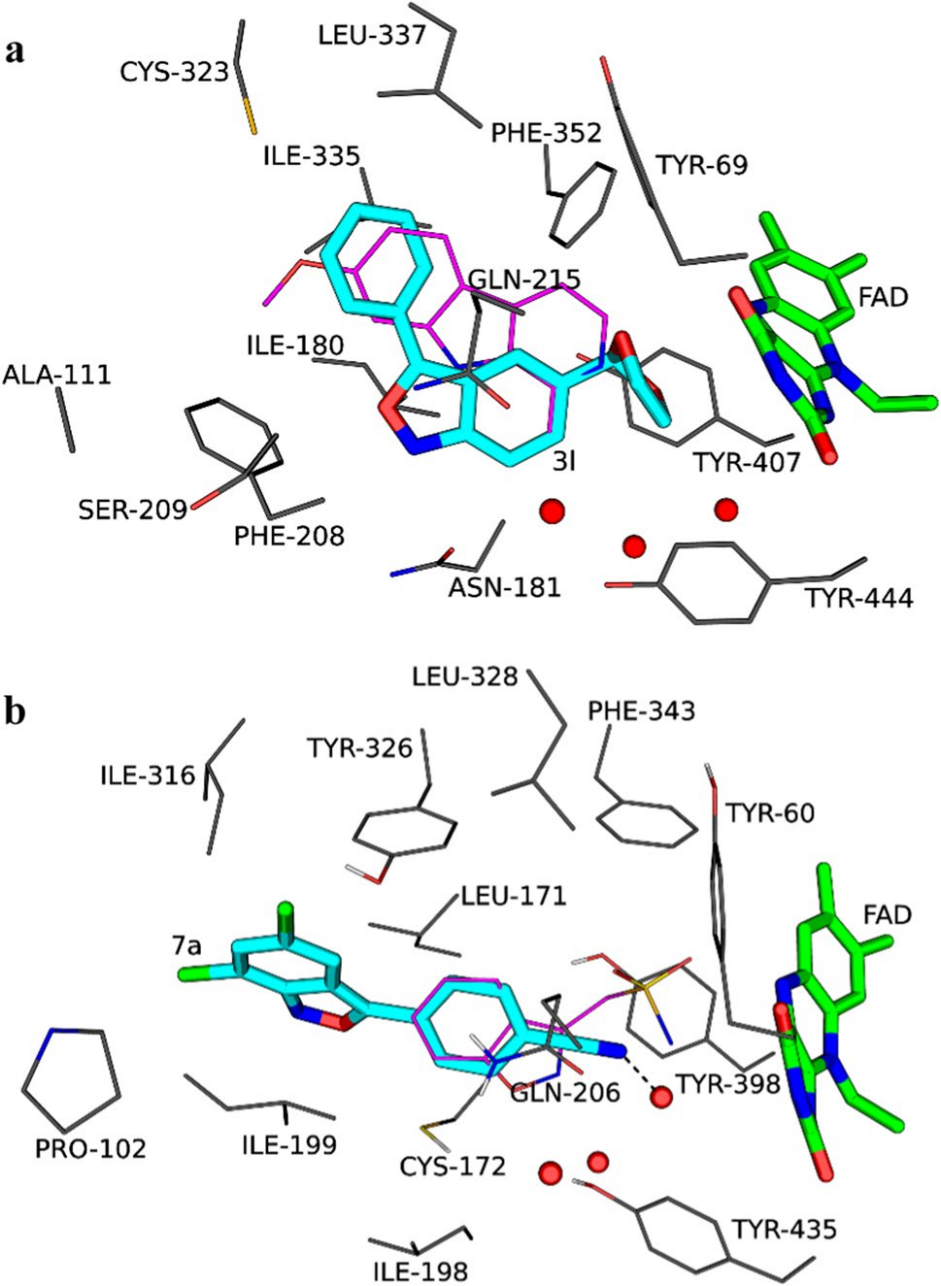
The predicted binding of **3l** (**a**) and **7a** (**b**) to MAO-A and MAO-B, respectively. The orientations of harmine in MAO-A and zonisamide in MAO-B are shown in magenta lines, while the FAD is shown in green sticks. Hydrogen bonding is indicated by the dashed lines

As mentioned, the X-ray crystal structure of zonisamide, a 1,2-benzisoxazole compound, in complex with MAO-B has been reported [19]. Zonisamide binds within the substrate cavity of the enzyme with the Ile-199 gating residue in the “open” conformation [20]. A molecular docking study was carried out with the Discovery Studio software according to the reported protocol with the aim of predicting the binding orientation of **7a** to MAO-B, using the structure of zonisamide bound to MAO-B (PDB code: 3PO7) [28]. The result shows that the binding of **7a** differs significantly from zonisamide with **7a** extending much deeper into the entrance cavity (Fig. 7). The 2,1-benzisoxazole ring thus binds within the entrance cavity where stabilization occurs via van der Waals interactions. Within the entrance cavity, the contribution of the chloro groups to inhibitor stabilization appears to be additive since **7a** is a more potent MAO-B inhibitor than **7b**, the mono-chloro substituted compound. The benzonitrile moiety is placed in the substrate cavity where the nitrile may undergo hydrogen bonding with a water molecule. This polar interaction by the nitrile may explain the finding that the *p*-benzonitrile substituted compounds **7a**–**c** were the most potent MAO-B inhibitors of this study. Other stabilizing interactions include π**···**π stacking and lp(S)**···**π interactions of both the phenyl and isoxazole with Tyr-326 and Cys-172, respectively, as well as a π**···**σ interaction the fused phenyl of the 2,1-benzisoxazole ring with Ile-199.

The binding of **3l**, a potent MAO-A inhibitor of this study, was also investigated by molecular docking, using the X-ray crystal structure of harmine bound to MAO-A (PDB code: 2Z5X) [28, 29]. Compound **3l** binds in the MAO-A active with the 1,3-dioxolanyl ring in proximity to the FAD, while the phenyl extends towards the entrance of the active site. While no hydrogen bonding is predicted, a lp(S)**···**π interaction between the phenyl substituent and Cys-323 may occur. Most interactions, however, are van der Waals interactions with active site residues.

## Conclusion

In conclusion, this study discovers three *p*-benzonitrile substituted compounds (**7a**–**c**) with submicromolar MAO-B inhibition potencies. While the 2,1-benzisoxazole ring contributes to inhibitor stabilization via π**···**π stacking, lp**···**π and van der Waals interactions, it does not seem to be a privileged structure for MAO-B inhibition. The 2,1-benzisoxazole ring, however, is suitable as a scaffold on which appropriate groups could be substituted. For MAO-B inhibition, *p*-benzonitrile substitution on the isoxazole ring and choro substitutions on C5 and C7 of the fused phenyl of the 2,1-benzisoxazole moiety are appropriate for potent MAO-B inhibition. 2,1-Benzisoxazole compounds such as **7a** may represent good leads for the future design of MAO-B specific inhibitors. To assess the possibility that **7a** might be developed as a drug, key physicochemical and pharmacokinetic properties were estimated with the SwissADME web tool provided by the Swiss Institute of Informatics (www.swissadme.ch) [30]. **7a** is predicted to have a log P of 3.89, to display moderate aqueous solubility, high absorption from the gastrointestinal track and to be able to penetrate the blood–brain barrier. Based on these calculations, **7a** could be an orally active MAO inhibitor with activity in the central nervous system, and this compound would therefore be a good candidate for preclinical studies.

## Experimental section

### Reagents and instrumentation

All reagents and solvents were obtained from commercial sources and were used without purification. DMSO was dried over molecular sieves (4 Å). Reactions were monitored by analytical thin layer chromatography (TLC) using Macherey–Nagel TLC sheets (Silufol UV-254) and the developed sheets were visualized under UV light. NMR spectra were recorded on Bruker AVANCE DPX 400 at 400 MHz and 101 MHz for ^1^H and ^13^C, respectively. Chemical shifts are reported as parts per million (δ, ppm) and were referenced to the residual solvent peaks for ^1^H spectra (7.26 ppm for CDCl_3_ and 2.50 ppm for DMSO-*d*_6_) and to carbon peaks of the solvent for ^13^C (39.52 ppm for DMSO-*d*_6_ and 77.16 ppm for CDCl_3_). Multiplicities are abbreviated as follows: s, singlet; d, doublet; t, triplet; q, quartet; m, multiplet; br, broad. Coupling constants, J, are reported in Hertz (Hz). Melting points were determined in open capillary tubes with an Electrothermal IA 9300 series digital melting point apparatus. High-resolution mass spectra (HRMS) were recorded with a Bruker maXis HRMS-ESI-QTOF instrument (ESI mode).

X-ray diffraction data were collected at a Rigaku SuperNova diffractometer using Cu–Kα (λ = 0.154184 nm) radiation. The structure has been solved with the ShelXT structure solution program using Intrinsic Phasing and refined with the ShelXL refinement package incorporated in the OLEX2 program package using Least Squares minimization [31–33]. The carbon-bound H atoms were placed in calculated positions. Empirical absorption correction was applied in the CrysAlisPro program complex (Agilent Technologies, 2014) using spherical harmonics, implemented in SCALE3 ABSPACK scaling algorithm. Supplementary crystallographic data have been deposited at Cambridge Crystallographic Data Centre: **2,220,356**. It can be obtained free of charge via www.ccdc.cam.ac.uk/data_request/cif (accessed on 18 November 2022).

For the MAO inhibition studies, a Varian Cary Eclipse fluorescence spectrophotometer was employed. Microsomes from insect cells containing recombinant human MAO-A and MAO-B (5 mg protein/mL) and kynuramine dihydrobromide were obtained from Sigma-Aldrich.

### Synthesis and characterization of 2,1-benzisoxazoles (3a–o, 5)

In a flat-bottom flask, a mixture of finely powdered sodium hydroxide (5 g, 0.125 mol) and isopropyl alcohol (40 mL) was stirred for 30 min. Arylacetonitrile (0.015 mol) and a *p*-substituted nitroarene (0.012 mol) were added sequentially to the flask. The reaction mixture was vigorously stirred at room temperature until the completion of the reaction (TLC monitoring, 8–24 h). The reaction was poured into 400 mL water and the precipitate that formed was removed by filtration and washed with water. The filtrate was treated with hydrochloric acid until the pH tested acidic, and the precipitate that formed was collected by filtration. The product was purified by recrystallization from isopropanol [22, 23].

### 5-Iodo-3-(4-methoxyphenyl)benzo[c]isoxazole (3a)

Orange solid, 61% (2.57 g) yield, m.p. 112–114 °C. ^1^H NMR (400 MHz, DMSO-*d*_6_) δ 8.53 (s, 1H), 8.09 (d, *J* = 8.7 Hz, 2H), 7.62 (d, *J* = 9.3 Hz, 1H), 7.48 (d, *J* = 9.3 Hz, 1H), 7.17 (d, *J* = 8.8 Hz, 2H), 3.88 (s, 3H) [34].

### 3-(4-Chlorophenyl)-5-iodobenzo[c]isoxazole (3b)

Brown solid, 71% (3.1 g) yield, m.p. 185–187 °C. ^1^H NMR (400 MHz, DMSO-*d*_6_) δ 8.59 (s, 1H), 8.17 (d, *J* = 8.6 Hz, 2H), 7.73—7.59 (m, 3H), 7.55 (d, *J* = 9.3 Hz, 1H) [24].

### 5-Chloro-3-(p-tolyl)benzo[c]isoxazole (3c)

Beige solid, 63% (1.86 g) yield, m.p. 136–138 °C.^1^H NMR (400 MHz, DMSO-*d*_6_) δ 8.22 (s, 1H), 8.02 (d, *J* = 8.2 Hz, 2H), 7.74 (d, *J* = 9.4 Hz, 1H), 7.48—7.40 (m, 3H), 2.42 (s, 3H) [24].

### 3-Phenyl-5-(phenylethynyl)benzo[c]isoxazole (3d)

Yellow solid, 57% (2.02 g) yield, m.p. 143–144 °C. ^1^H NMR (400 MHz, DMSO-*d*_6_) δ 8.39 (s, 1H), 8.19 (d, *J* = 6.6 Hz, 2H), 7.76 (d, *J* = 9.2 Hz, 1H), 7.79—7.61 (m, 5H), 7.59—6.71 (m, 4H). ^13^C NMR (101 MHz, DMSO-*d*_6_) δ 165.0, 156.6, 134.1, 131.9, 131.5, 130.1, 129.5, 129.3, 127.5, 127.2, 125.3, 122.5, 119.3, 116.1, 114.2, 91.0, 89.8.^.^HRMS (ESI) calcd for C_21_H_13_NO [M + Na]^+^ 357.9699, found 357.9696 [24].

### 5-Chloro-6-(5-methyl-1,2,4-oxadiazol-3-yl)-3-phenylbenzo[c]isoxazole (3e)

Beige solid, 72% (2.68 g) yield, m.p. 171–172 °C. ^1^H NMR (400 MHz, DMSO-*d*_6_) δ 8.52 (s, 1H), 8.22 (d, *J* = 22.5 Hz, 3H), 7.71 – 7.62 (m, 3H), 2.74 (s, 3H). ^13^C NMR (101 MHz DMSO-*d*_6_) δ 177.8, 166.7, 165.5, 155.7, 131.8, 130.2, 130.0, 127.7, 127.3, 127.1, 123.0, 120.2, 114.4, 12.4.^.^HRMS (ESI) calcd for C_16_H_10_ClN_3_O_2_ [M + Na]^+^ 334.0354, found 334.0350.

### 3-(3-Chlorophenyl)-5-(2-methyl-1,3-dioxolan-2-yl)benzo[c]isoxazole (3f)

Beige solid, 58% (2.19 g) yield, m.p. 175–177 °C. ^1^H NMR (400 MHz, DMSO-*d*_6_) δ 8.11—8.05 (m, 2H), 7.93 (t, *J* = 1.2 Hz, 1H), 7.72—7.65 (m, 2H), 7.65—7.58 (m, 1H), 7.49 (dd, *J* = 9.3, 1.5 Hz, 1H), 4.09—3.98 (m, 2H), 3.87–3.74 (m, 2H), 1.64 (s, 3H). ^13^C NMR (101 MHz, DMSO-*d*_6_) δ 164.79, 157.59, 140.35, 131.24, 130.69, 130.19, 127.85, 126.98, 116.23, 115.78, 113.74, 108.23, 64.72, 26.90. HRMS (ESI) calcd for C_17_H_14_ClNO_3_ [M + Na]^+^ 304.0944, found 304.0950 [24].

### 5-Bromo-3-phenylbenzo[c]isoxazole (3g)

Yellow solid, 74% (3.04 g) yield, m.p. 116–118 °C. ^1^H NMR (400 MHz, DMSO-*d*_6_) δ 8.42 (s, 1H), 8.15 (dd, *J* = 7.9, 1.6 Hz, 2H), 7.70—7.64 (m, 1H), 7.64—7.56 (m, 3H), 7.55 (dd, *J* = 9.4, 1.6 Hz, 1H) [35].

### 5-Iodo-3-phenylbenzo[c]isoxazole (3h)

Yellow solid, 67% (3.2 g) yield, m.p. 112–115 °C. ^1^H NMR (400 MHz, DMSO-*d*_6_) δ 8.58 (s, 1H), 8.13 (dd, *J* = 7.7, 1.6 Hz, 2H), 7.65—7.63 (m, 1H), 7.63—7.47 (m, 3H), 7.54 (d, *J* = 9.3 Hz, 1H) [35].

### 5-Iodo-3-(p-tolyl)benzo[c]isoxazole (3i)

Beige solid, 78% (3.12 g) yield, m.p. 109–110 °C. ^1^H NMR (400 MHz, DMSO-*d*_6_) δ 8.56 (s, 1H), 8.11 – 7.98 (m, 3H), 7.64 (d, *J* = 9.3 Hz, 1H), 7.51 (d, *J* = 9.3 Hz, 1H), 7.44 (d, *J* = 8.0 Hz, 2H), 2.42 (s, 3H). ^13^C NMR (101 MHz, DMSO-*d*_6_) δ 163.8, 156.1, 141.6, 134.0, 139.2, 130.6, 129.9, 127.0, 125.4, 124.8, 117.2, 115.9, 21.6. HRMS (ESI) calcd for C_14_H_10_INO [M + Na]^+^ 357.9699, found 357.9687.

### 5-Chloro-3-phenylbenzo[c]isoxazole (3j)

Yellow solid, 76% (4.2 g) yield, m.p. 115–117 °C. ^1^H NMR (400 MHz, DMSO-*d*_6_) δ 8.25 (s, 1H), 8.13 (d, *J* = 6.6 Hz, 2H), 7.77 (d, *J* = 9.4 Hz, 1H), 7.69—7.52 (m, 3H), 7.45 (dd, *J* = 9.5, 1.5 Hz, 1H) [36].

### 6-Dichloro-3-phenylbenzo[c]isoxazole (3k)

Beige solid, 64% (2.03 g) yield, m.p. 157–159 °C. ^1^H NMR (400 MHz, DMSO-*d*_6_) δ 8.55 (s, 1H), 8.20 (s, 1H), 8.16 (dd, *J* = 7.4, 2.1 Hz, 2H), 7.71—7.58 (m, 3H). ^13^C NMR (101 MHz, DMSO-*d*_6_) δ 165.4, 156.3, 135.4, 131.8, 130.1, 128.5, 127.3, 127.0, 122.8, 116.7, 113.1. HRMS (ESI) calcd for C_13_H_7_Cl_2_NO [M + Na]^+^ 285.9797, found 285.9799 [24].

### 5-(1,3-Dioxolan-2-yl)-3-phenylbenzo[c]isoxazole (3l)

Beige solid, 62% (1.98 g) yield, m.p. 137–138 °C. ^1^H NMR (400 MHz, DMSO-*d*_6_) δ 8.17—8.08 (m, 3H), 7.75—7.63 (m, 3H), 7.63 – 7.56 (m, 1H), 7.46 (d, *J* = 9.3 Hz, 1H), 5.82 (s, 1H), 4.20—4.04 (m, 2H), 4.04—3.93 (m, 2H). ^13^C NMR (101 MHz, DMSO-*d*_6_) δ 165.1, 158.0, 135.5, 131.4, 130.5, 130.1, 127.7, 127.0, 120.1, 115.9, 113.6, 103.1, 65.6.^.^HRMS (ESI) calcd for C_16_H_13_NO_3_ [M + Na]^+^ 304.0944, found 304.0950 [37].

### 3-(4-Bromophenyl)-5-chlorobenzo[c]isoxazole (3m)

Beige solid, 76% (1.40 g) yield, m.p. 230–232 °C. ^1^H NMR (400 MHz, DMSO-*d*_6_) δ 8.24 (s, 1H), 8.06 (t, *J* = 19.0 Hz, 2H), 7.98—7.59 (m, 3H), 7.46 (d, *J* = 9.5 Hz, 1H) [34].

### 5-Chloro-3-(4-chlorophenyl)benzo[c]isoxazole (3n)

White solid, 73% (1.26 g) yield, m.p. 229–232 °C. ^1^H NMR (400 MHz, DMSO-*d*_6_) δ 8.23 (s, 1H), 8.19—8.07 (m, 2H), 7.77 (dd, J = 12.6, 3.1 Hz, 1H), 7.76—7.57 (m, 2H), 7.49—7.40 (m, 1H).^13^C NMR (101 MHz, DMSO-*d*_6_) δ 163.4, 156.4, 136.2, 133.2, 130.4, 130.1, 128.7, 126.3, 119.7, 117.7, 114.6.^.^HRMS (ESI) calcd for C_13_H_7_Cl_2_NO [M + Na]^+^ 285.9803, found 285.9798 [24].

### 5-Chloro-3-(4-methoxyphenyl)benzo[c]isoxazole (3o)

Yellow solid, 68% (2.06 g) yield, m.p. 174 °C. ^1^H NMR (400 MHz, CDCl_3_) δ 7.91 (s, 1H), 7.82 (d, *J* = 29.8 Hz, 1H), 7.54 (d, *J* = 9.4 Hz, 1H), 7.32—7.14 (m, 3H), 7.03 (t, *J* = 19.9 Hz, 1H), 4.15—3.66 (m, 2H) [22].

### 5,5'-Oxybis(3-phenylbenzo[c]isoxazole) (5)

White solid, 63% (3.05 g) yield, m.p. 190–192 °C. ^1^H NMR (400 MHz, DMSO-*d*_6_) δ 8.02 (d, *J* = 7.5 Hz, 4H), 7.81 (d, *J* = 9.6 Hz, 2H), 7.56 (dd, *J* = 16.8, 9.6 Hz, 7H), 7.41 (d, *J* = 9.5 Hz, 2H), 7.41 (d, *J* = 9.5 Hz, 1H) [38].

### Synthesis and characterization of 2,1-benzisoxazoles (7a–c)

In a flat-bottom flask, a mixture of finely powdered sodium hydroxide (5 g, 0.125 mol) and isopropyl alcohol (40 mL) was stirred for 30 min. 1-(4-Cyanobenzyl)-1*H*-1,2,4-triazole (0.015 mol) and a *p*-substituted nitroarene (0.012 mol) were added sequentially to the flask. The reaction mixture was vigorously stirred at room temperature until the completion of the reaction (TLC monitoring, 20 h). The reaction was poured into 400 mL water and the precipitate that formed was removed by filtration and washed with water. The filtrate was treated with hydrochloric acid until the pH tested acidic, and the precipitate that formed was collected by filtration. The product was purified by recrystallization from isopropanol, and then from N,N-dimethylformamide.

### 4-(5,7-Dichlorobenzo[c]isoxazol-3-yl)benzonitrile (7a)

Light yellow solid, 53% (0.77 g) yield, m.p. 240–241 °C. ^1^H NMR (400 MHz, DMSO-*d*_6_) δ 8.33 (d, *J* = 8.4 Hz, 3H), 8.07 (d, *J* = 8.4 Hz, 2H), 7.81 (d, *J* = 17.6 Hz, 1H). ^13^C NMR (101 MHz, DMSO-*d*_6_) δ 164.4, 154.9, 133.9, 132.3, 130.6, 127.9, 121.9, 119.3, 118.7, 116.3, 113.8. HRMS (ESI) calcd for C_14_H_6_Cl_2_N_2_O [M + Na]^+^ 310.9749, found 310.9740.

### 4-(5-Chlorobenzo[c]isoxazol-3-yl)benzonitrile (7b)

Yellow solid, 67% (2.05 g) yield, m.p. 236–237 °C. ^1^H NMR (400 MHz, DMSO-*d*_6_) δ 8.33 (d, *J* = 1.6 Hz, 3H), 8.07 (s, 2H), 7.82 (s, 1H), 7.49 (s, 1H). ^13^C NMR (101 MHz, DMSO-*d*_6_) δ 162.4, 156.4, 133.9, 133.5, 131.3, 131.0, 127.7, 119.8, 118.8, 118.0, 115.6, 113.3. HRMS (ESI) calcd for C_14_H_7_ClN_2_O [M + Na]^+^ 277.0139, found 277.0133.

### 4-(5-Chloro-6-(5-methyl-1,2,4-oxadiazol-3-yl)benzo[c]isoxazol-3-yl)benzonitrile (7c)

Yellow solid, 65% (2.62 g) yield, m.p. 257–258 °C. ^1^H NMR (400 MHz, DMSO-*d*_6_) δ 8.58 (s, 1H), 8.38 (d, *J* = 7.4 Hz, 2H), 8.30 (s, 1H), 8.09 (d, *J* = 8.1 Hz, 2H). ^13^C NMR (101 MHz, DMSO-*d*_6_) δ 164.3, 155.0, 133.9, 132.3, 130.6, 127.9, 121.9, 119.3, 118.7, 116.3, 113.8. HRMS (ESI) calcd for C_17_H_9_ClN_4_O_2_ [M + Na]^+^ 359.0306, found 359.0301.

### MAO inhibition—determination of IC_50_ values

IC_50_ values for the inhibition of MAO were measured as described previously [28]. Microsomes from insect cells containing recombinant human MAO-A and MAO-B (5 mg protein/mL) served as enzyme sources (Sigma-Aldrich) while kynuramine was used as non-specific MAO substrate. The enzyme reactions (200 µL reactions prepared in 96-well microtiter plates) contained phosphate buffer (100 mM, pH 7.4), 50 µM kynuramine and the test inhibitors at concentrations of 0.003–100 µM. Stock solutions of the test inhibitors were dissolved in DMSO and added to the enzyme reactions to give a final DMSO concentration of 4% (v/v). The reactions were initiated with addition of MAO-A (0.0075 mg protein/mL) or MAO-B (0.015 mg protein/mL) and after 20 min of incubation at 37 °C, the reactions were terminated by the addition of 80 µL NaOH (2 N). The concentration of 4-hydroxyquinoline, the product of kynuramine oxidation by MAO, was measured by fluorescence spectrophotometry (λ_ex_ = 310 nm; λ_em_ = 400 nm). IC_50_ values were estimated from sigmoidal plots of enzyme catalytic rate versus the inhibitor concentration (log[I]) which were constructed with the Prism software package (GraphPad). IC_50_ values were estimated in triplicate determinations from these plots and are expressed as mean ± standard deviation (SD). Global (shared) fitting of the kinetic data yielded the following K_m_ and V_max_ values for kynuramine: K_m_ = 49.8 ± 3.07 and 32.4 ± 3.73 µM for MAO-A and MAO-B, respectively; V_max_ = 34.0 ± 0.80 and 8.39 ± 0.28 nmol/min.mg protein, for MAO-A and MAO-B, respectively.

### MAO inhibition—Lineweaver–Burk plots

For each of the selected test inhibitors, a set of six Lineweaver–Burk plots were constructed using the following inhibitor concentrations: 0 × IC_50_, ¼ × IC_50_, ½ × IC_50_, ¾ × IC_50_, 1 × IC_50_ and 1¼ × IC_50_. For each line, the concentration of kynuramine ranged from 15–250 µM. The reactions were carried out as described above for the IC_50_ value determinations with the exception that the final MAO concentrations in the reactions were 0.015 mg protein/mL for both MAO-A and MAO-B. Linear regression was carried out with the Prism software package.

### Molecular docking

Molecular docking was carried out with the Discovery Studio 3.1 software package as described previously [28]. The X-ray crystal structures of MAO-A (PDB code: 2Z5X) and MAO-B (PDB code: 3PO7) with harmine and zonisamide, respectively, bound to the active sites were selected as protein models [19, 29]. Illustrations were prepared with the PyMOL molecular graphics system [39].

### Supplementary Information

Below is the link to the electronic supplementary material.

Supplementary file1 (PDF 1805 KB)
1H NMR and 13C NMR spectra for the synthesised compounds and crystallographic data for 3c.
