# Supplementary material for: Monoamine oxidase inhibition properties of 2,1-benzisoxazole derivatives
Source: Mol Divers. 2023 Mar 19;28(3):1009–21. doi: 10.1007/s11030-023-10628-4 (PMC11269473; doi:10.1007/s11030-023-10628-4)

SUPPLEMENTARY MATERIAL – MOLECULAR DIVERSITY

Monoamine oxidase inhibition properties of 2,1-benzisoxazole derivatives

Anton Shetnev [1], Alexandr Kotov [1], Anna Kunichkina [1], Irina Proskurina [1], Sergey Baykov [2], Michail Korsakov [1], Anél Petzer [3], Jacobus P. Petzer [3,*]

[1] *Pharmaceutical Technology Transfer Center, Yaroslavl State Pedagogical University named after K.D. Ushinsky, 108 Respublikanskaya St., Yaroslavl, 150000, Russian Federation*

[2] *Institute of Chemistry, Saint Petersburg State University, 7/9 Universitetskaya Nab., Saint Petersburg, 199034, Russian Federation*

[3] *Pharmaceutical Chemistry and Centre of Excellence for Pharmaceutical Sciences, North-West University, Potchefstroom 2520, South Africa*

*Corresponding author.
E-mail address: jacques.petzer@nwu.ac.za

Crystal data of 3c

Table S1. Crystal data and structure refinement for **3c**

Empirical formula	$C_{14}H_{10}ClNO$
Formula weight	243.68
Temperature/K	100(2)
Crystal system	triclinic
Space group	P-1
a/Å	6.8148(4)
b/Å	7.0848(3)
c/Å	11.9088(5)
α/°	87.506(4)
β/°	83.896(4)
γ/°	81.141(4)
Volume/Å^3	564.68(5)
Z	2
ρcalcg/cm^3	1.433
μ/mm^{-1}	2.827
F(000)	252.0
Crystal size/mm^3	0.18 × 0.13 × 0.11
Radiation	CuKα (λ = 1.54184)
2Θ range for data collection/°	7.468 to 136.7
Index ranges	$-8 \leq h \leq 8$, $-8 \leq k \leq 8$, $-12 \leq l \leq 14$
Reflections collected	8158
Independent reflections	2047 [R_{int} = 0.0357, R_{sigma} = 0.0319]
Data/restraints/parameters	2047/0/155
Goodness-of-fit on F^2	1.063
Final R indexes [I>=2σ (I)]	R_1 = 0.0393, wR_2 = 0.1028
Final R indexes [all data]	R_1 = 0.0464, wR_2 = 0.1094
Largest diff. peak/hole / e Å^{-3}	0.46/–0.30
**CCDC**	**2220356**

^{1}H NMR spectra of 5-iodo-3-(4-methoxyphenyl)benzo[c]isoxazole **(3a)**

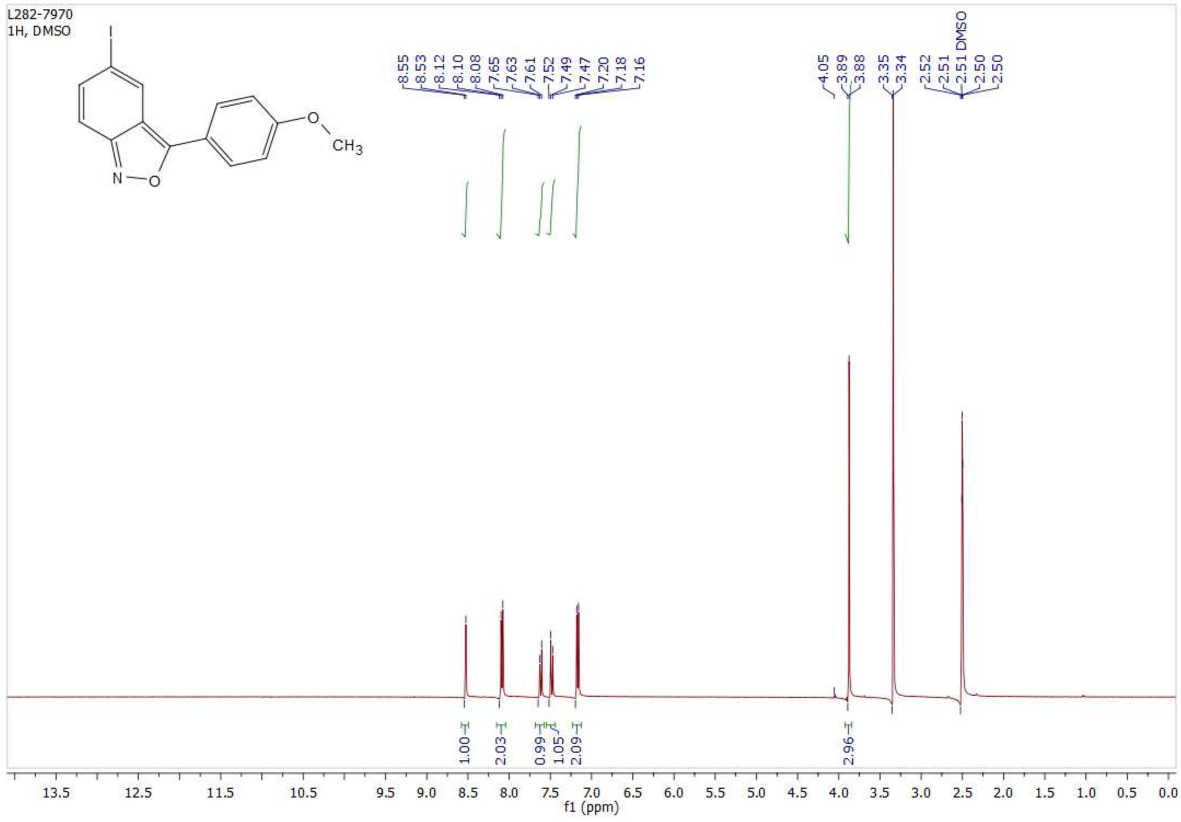

^{1}H NMR spectra of 3-(4-chlorophenyl)-5-iodobenzo[c]isoxazole **(3b)**

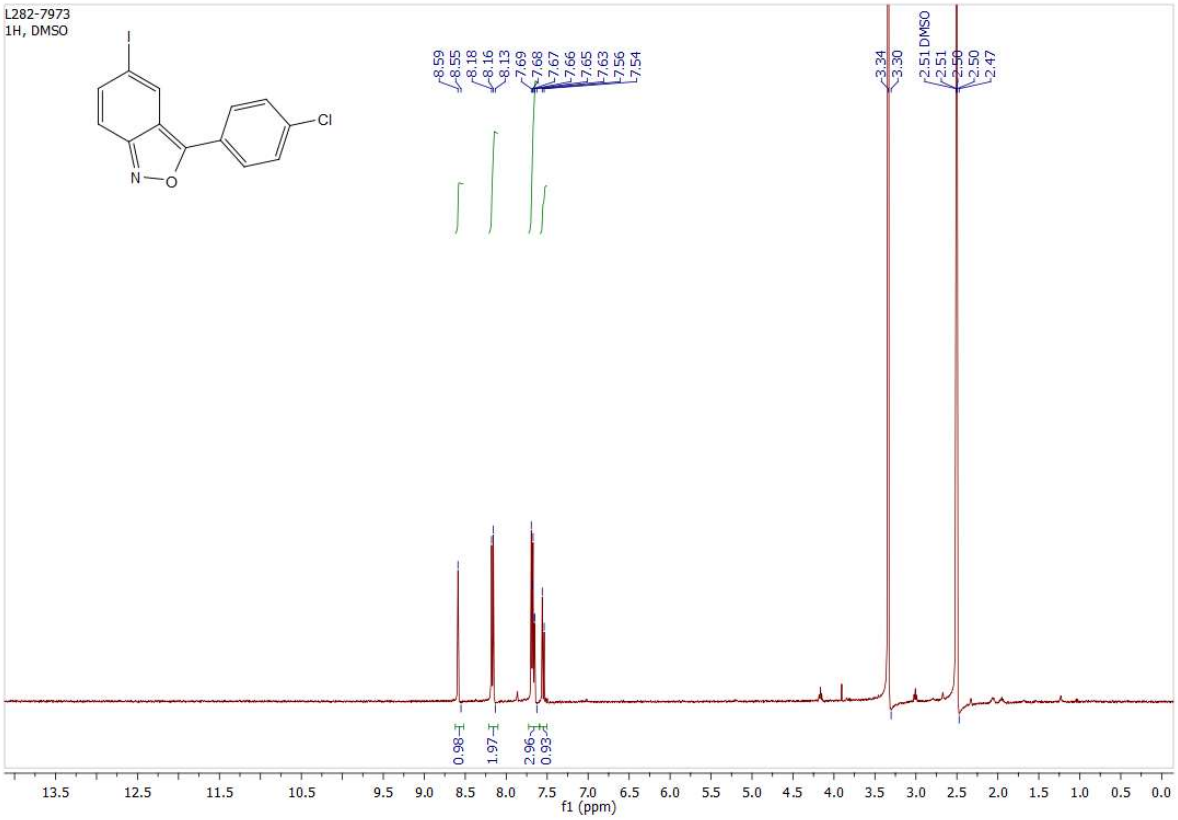

^{1}H NMR spectra of 5-chloro-3-(p-tolyl)benzo[c]isoxazole **(3c)**

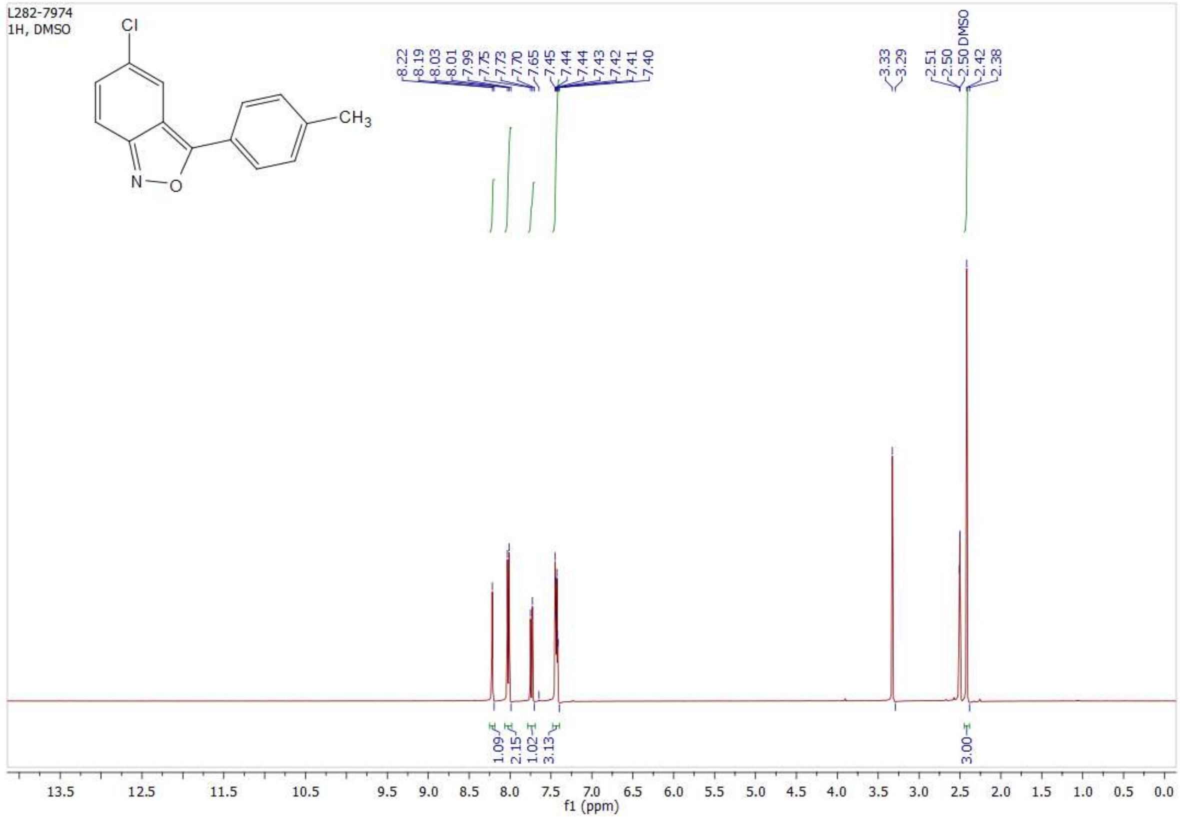

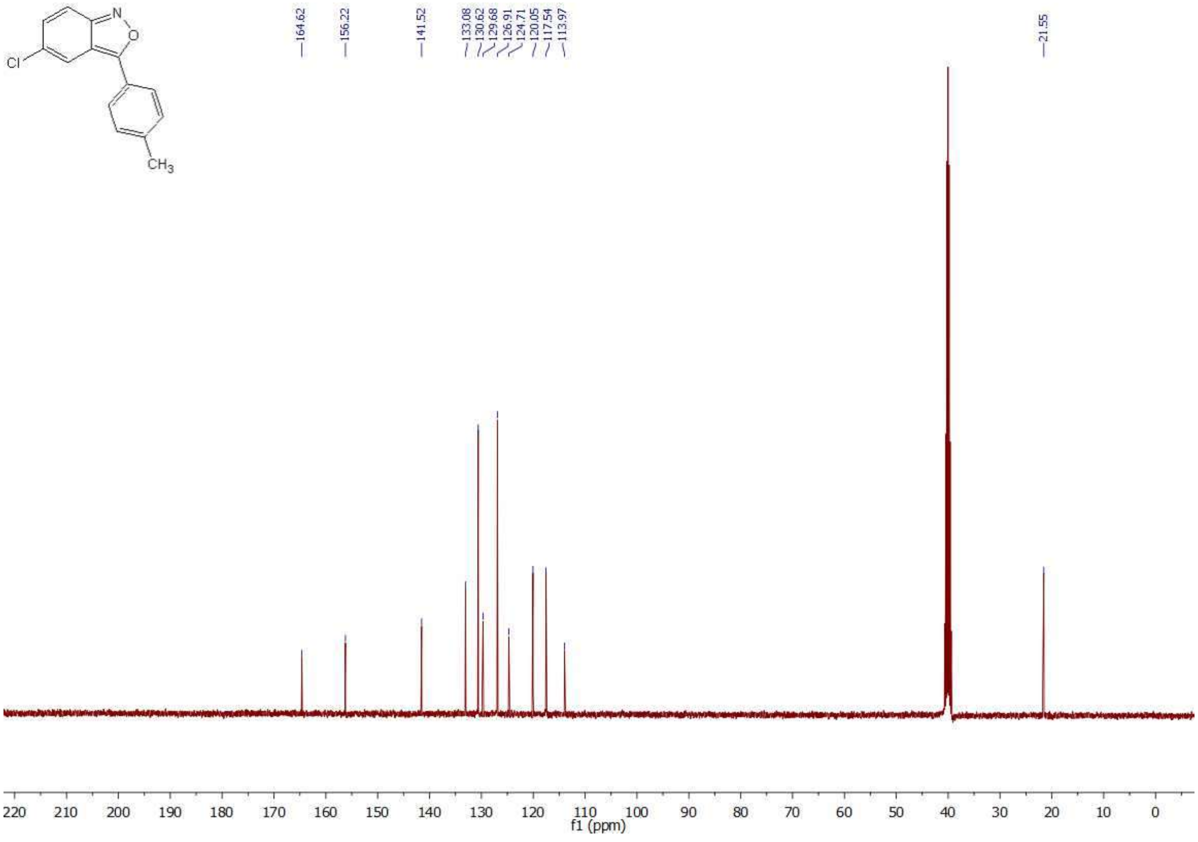

^{1}H NMR spectra of 3-phenyl-5-(phenylethynyl)benzo[c]isoxazole **(3d)**

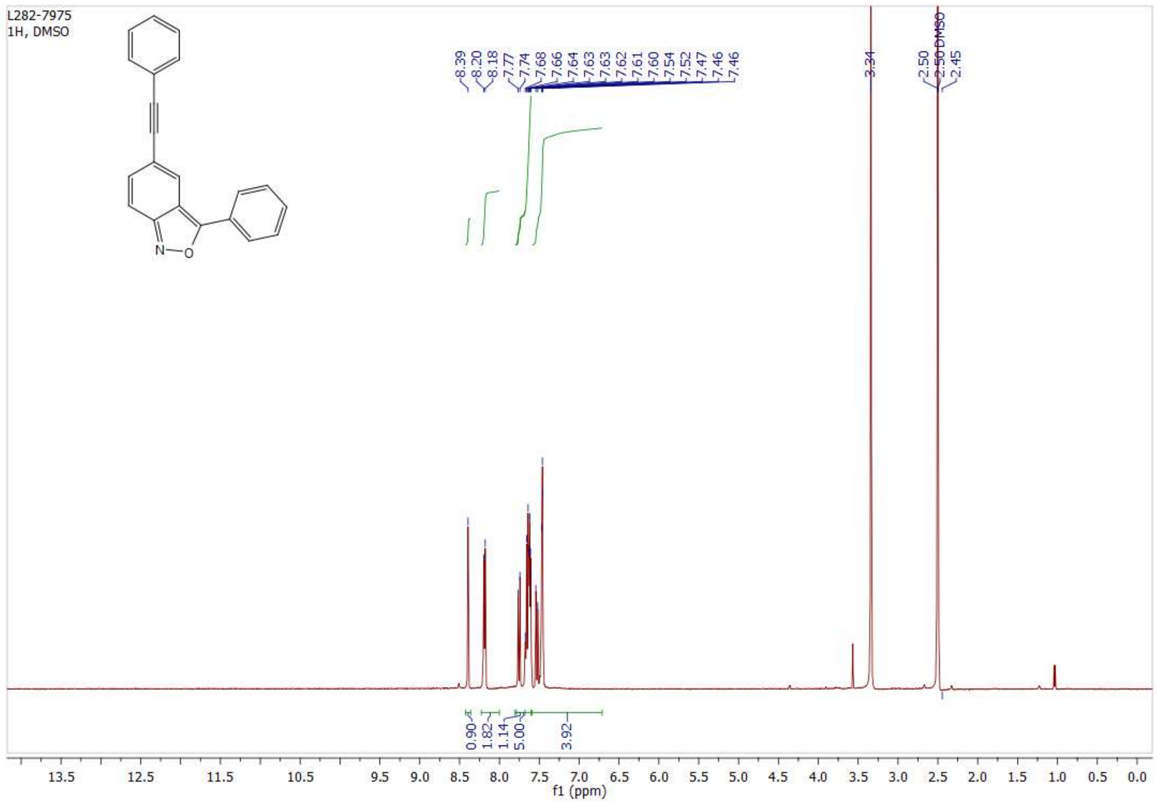

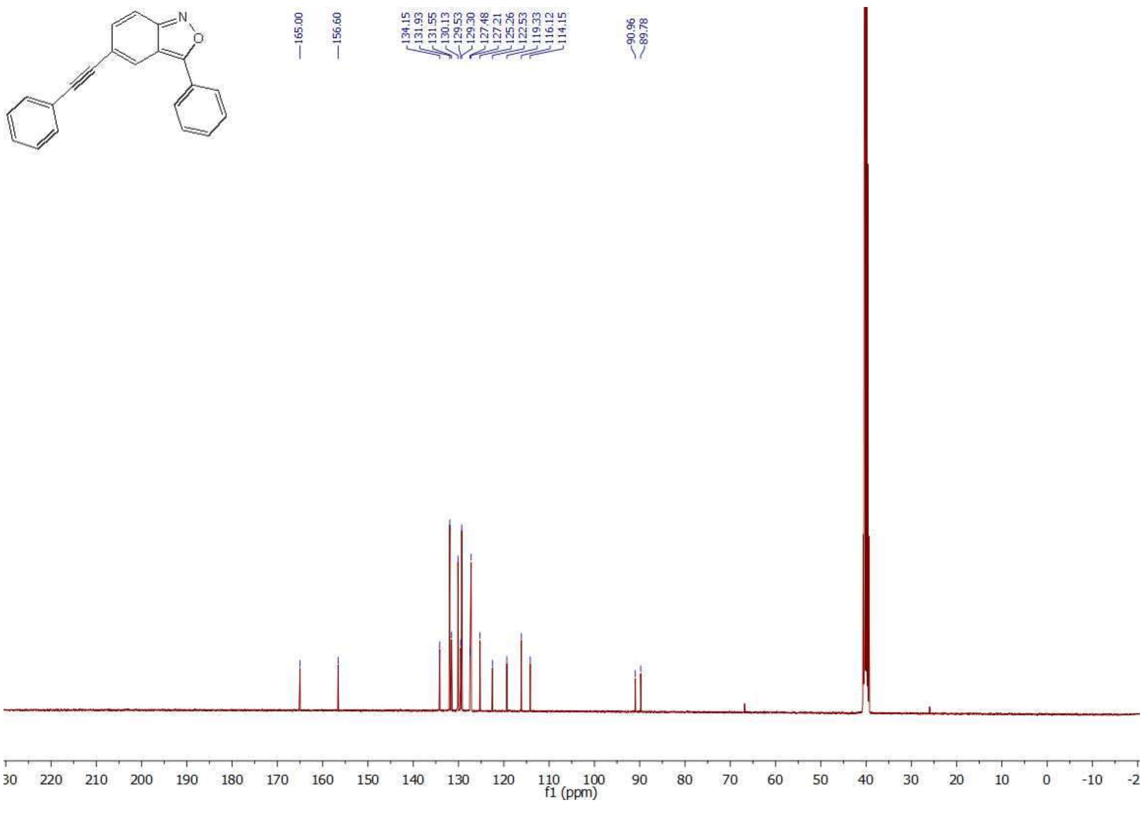

^{1}H NMR spectra of 5-chloro-6-(5-methyl-1,2,4-oxadiazol-3-yl)-3-phenylbenzo[c]isoxazole **(3e)**

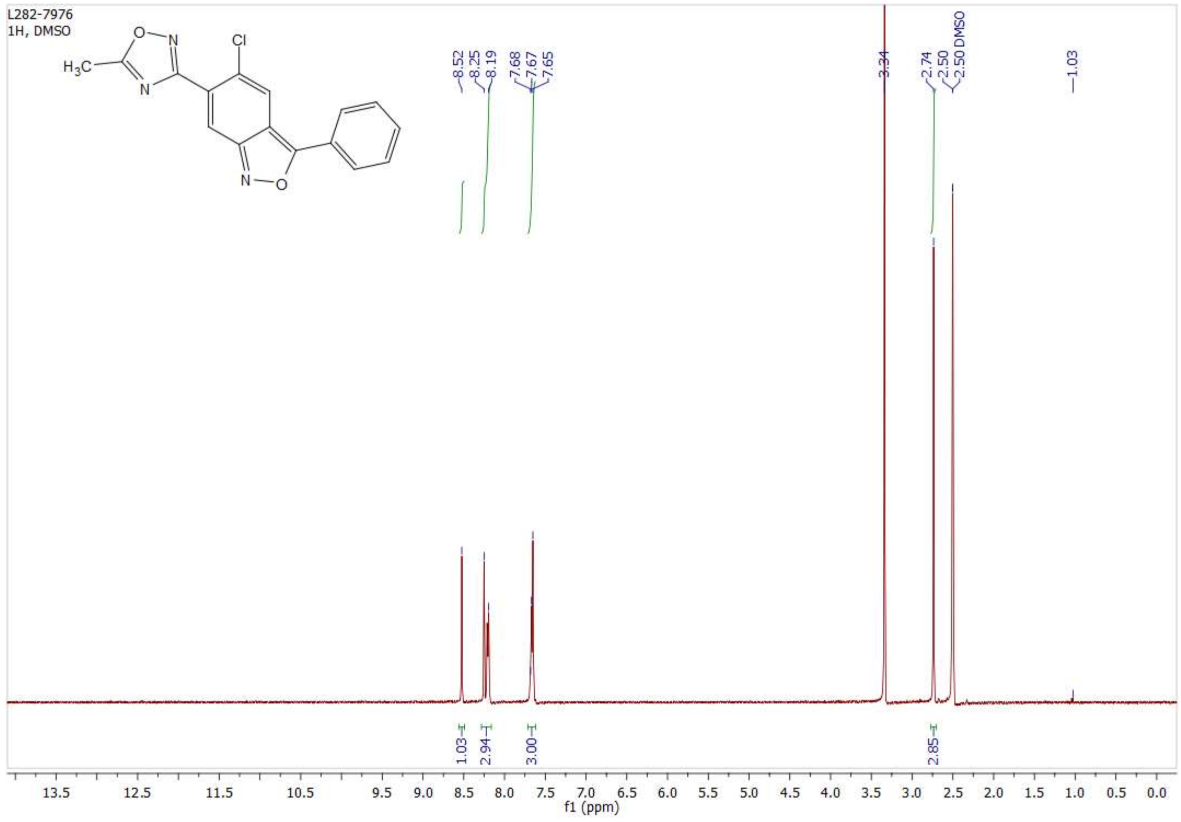

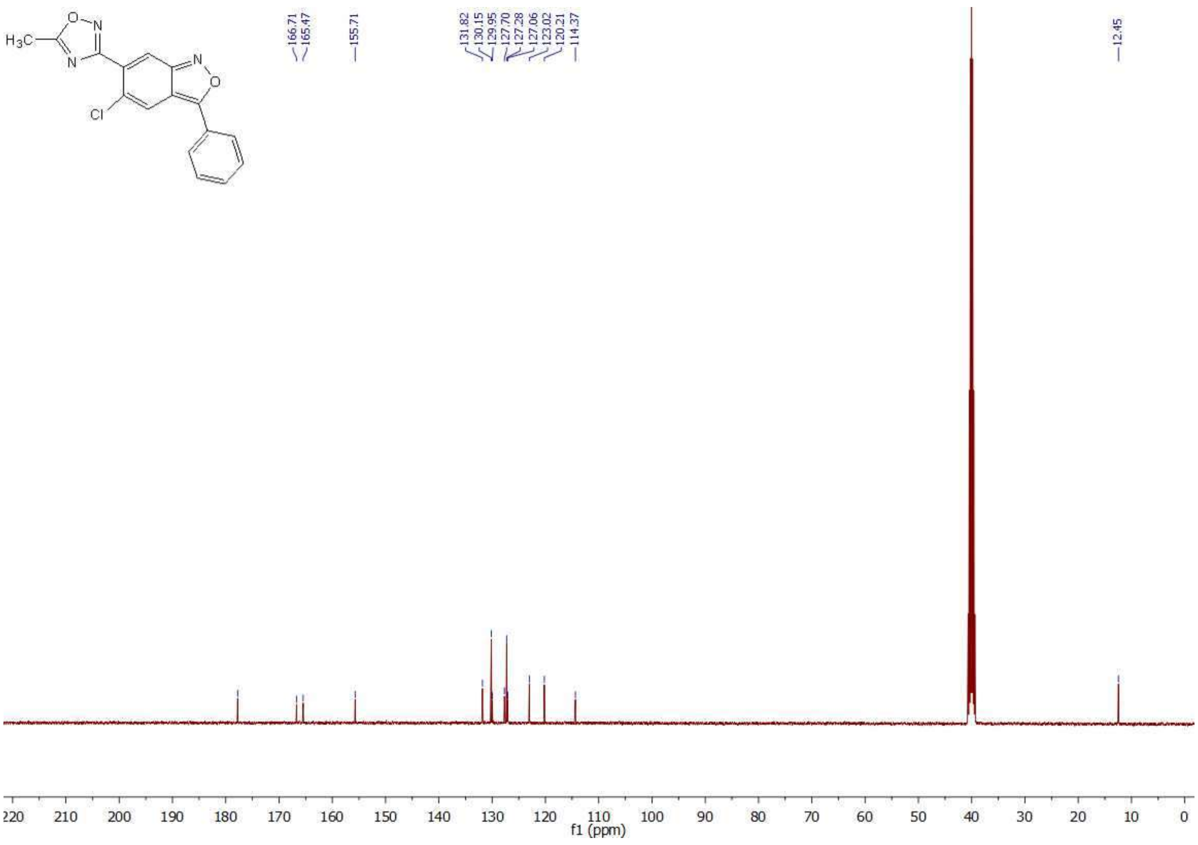

^{1}H NMR spectra of 3-(3-chlorophenyl)-5-(2-methyl-1,3-dioxolan-2-yl)benzo[c]isoxazole **(3f)**

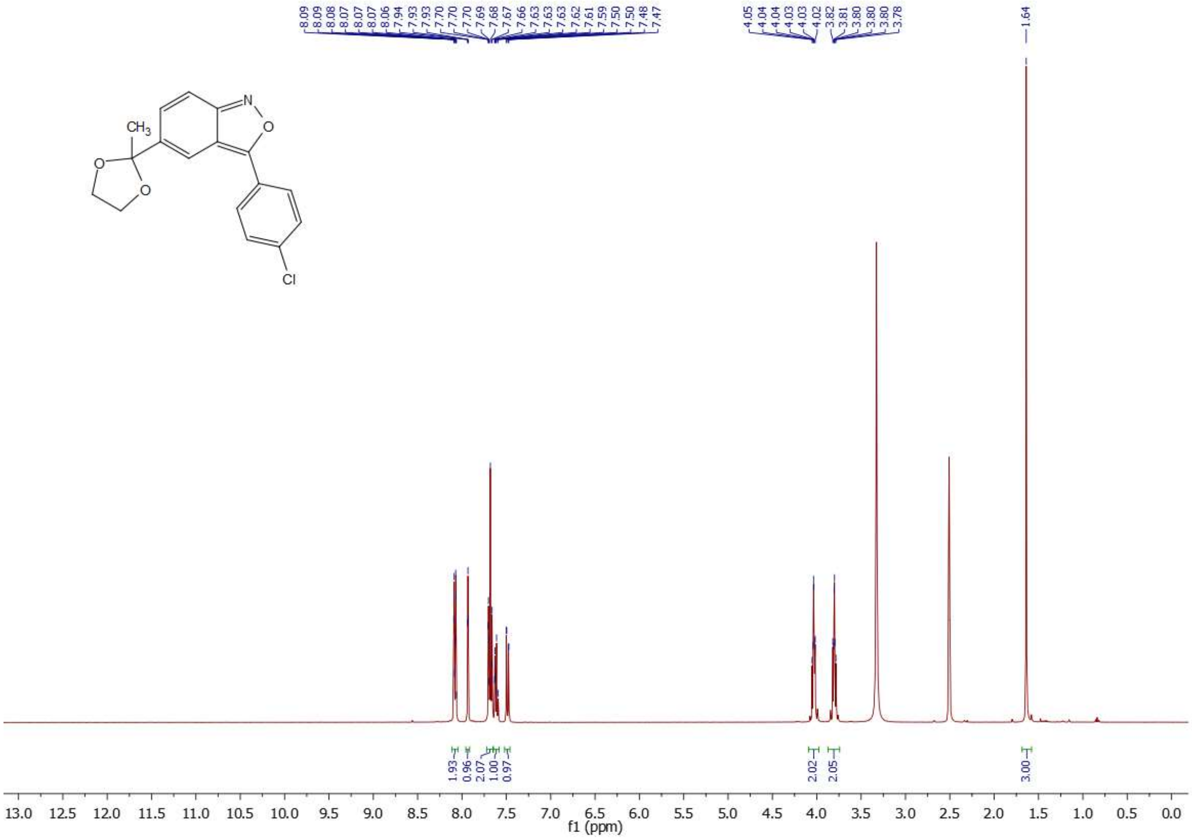

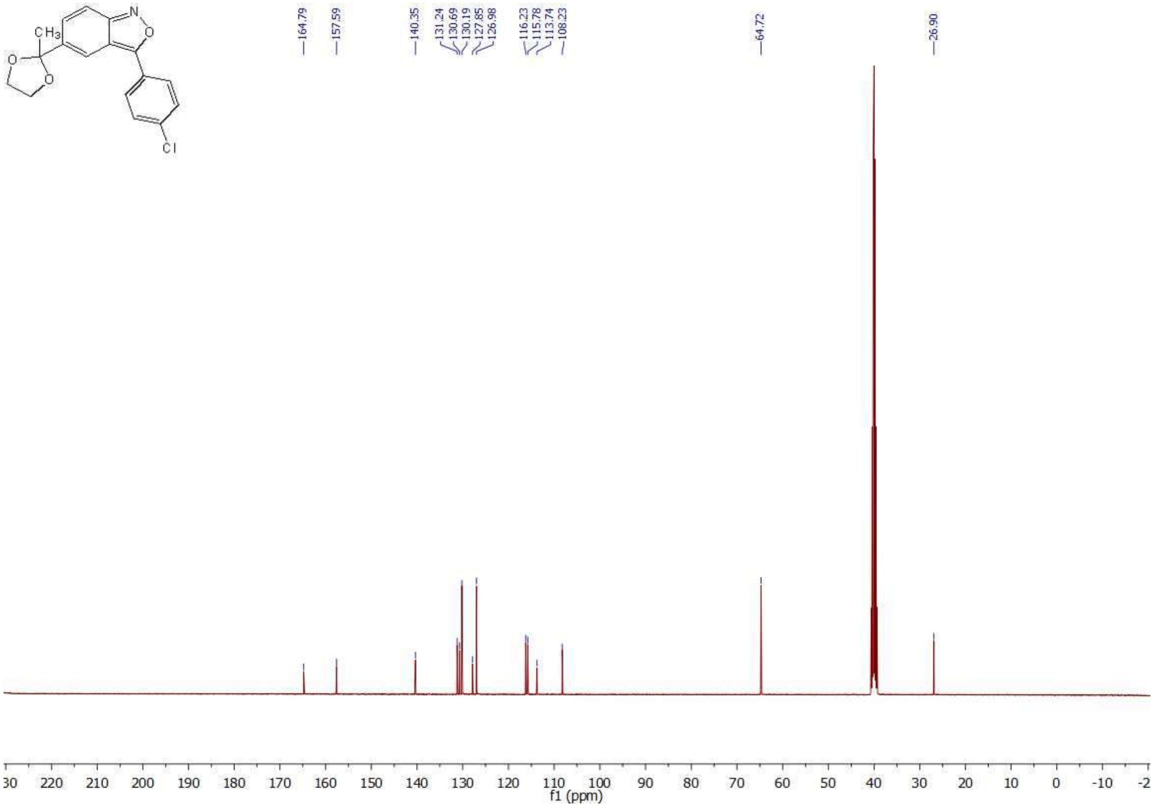

^{1}H NMR spectra of 5-bromo-3-phenylbenzo[c]isoxazole **(3g)**

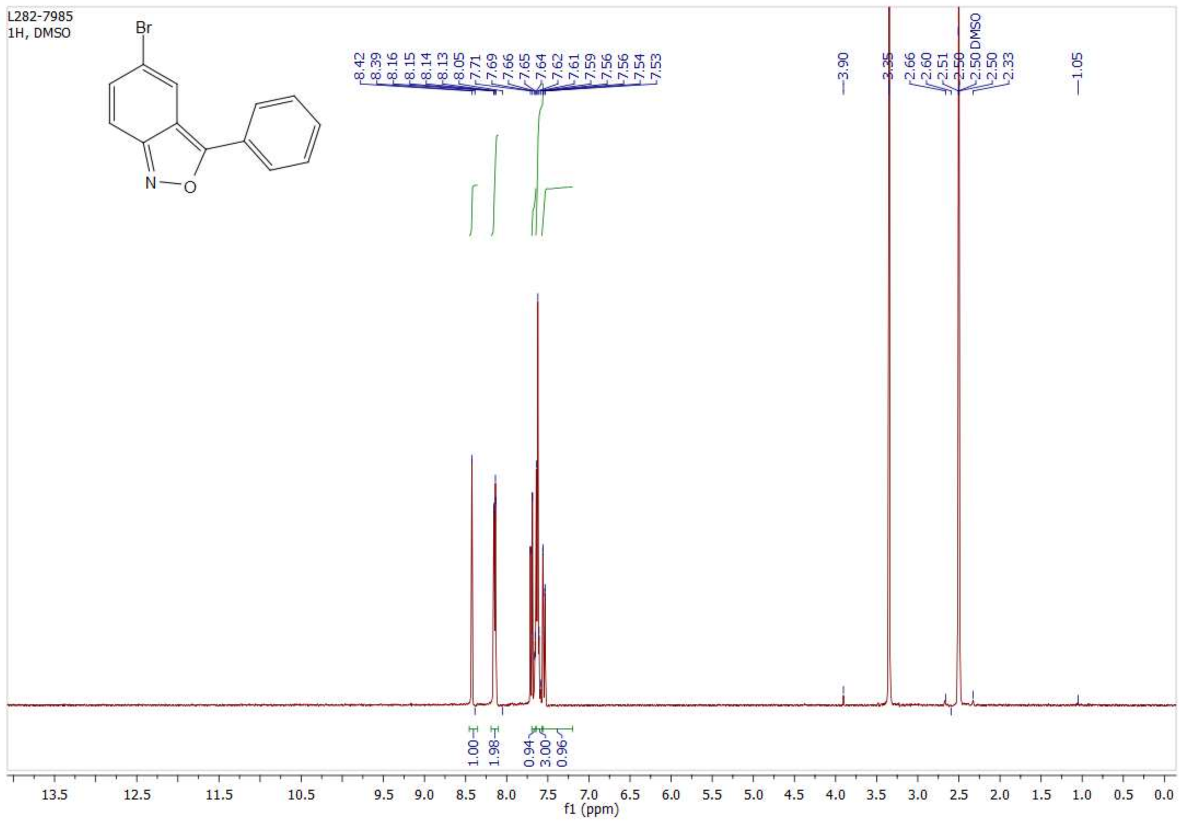

^{1}H NMR spectra of 5-iodo-3-phenylbenzo[c]isoxazole **(3h)**

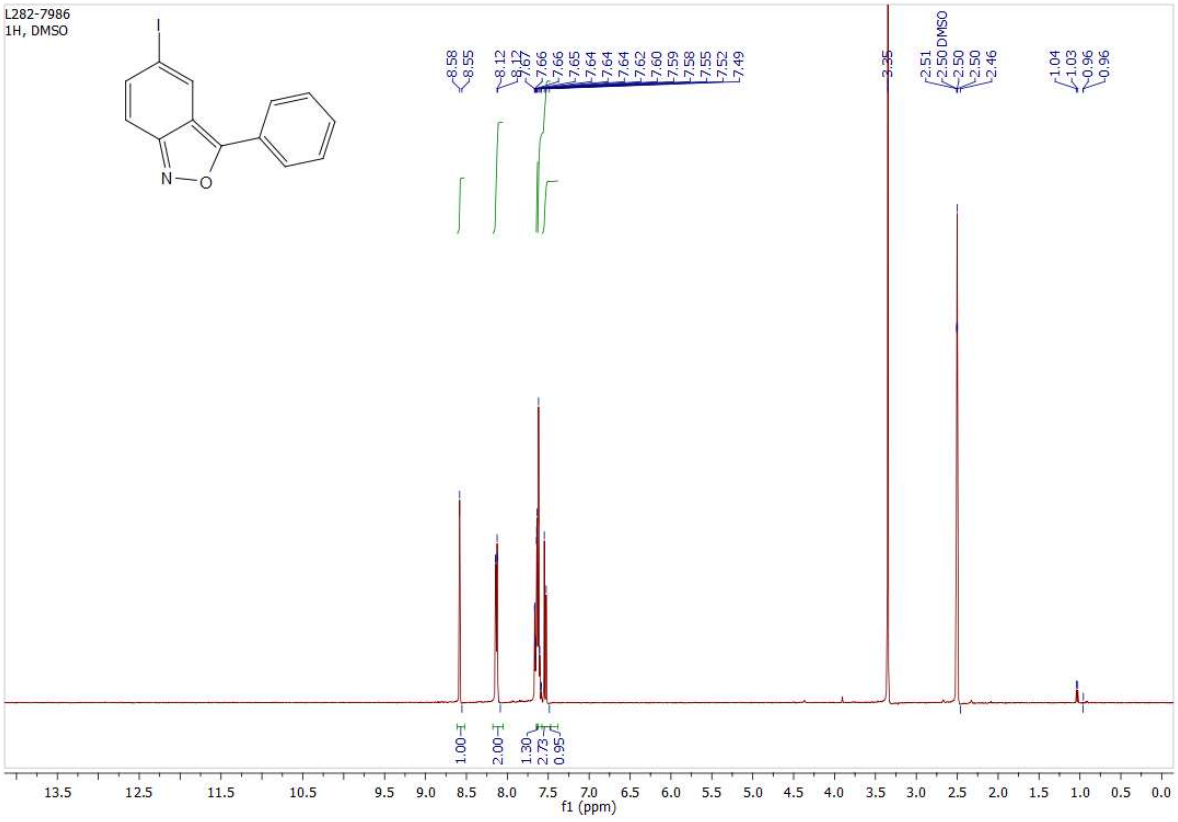

^{1}H NMR spectra of 5-iodo-3-(p-tolyl)benzo[c]isoxazole **(3i)**

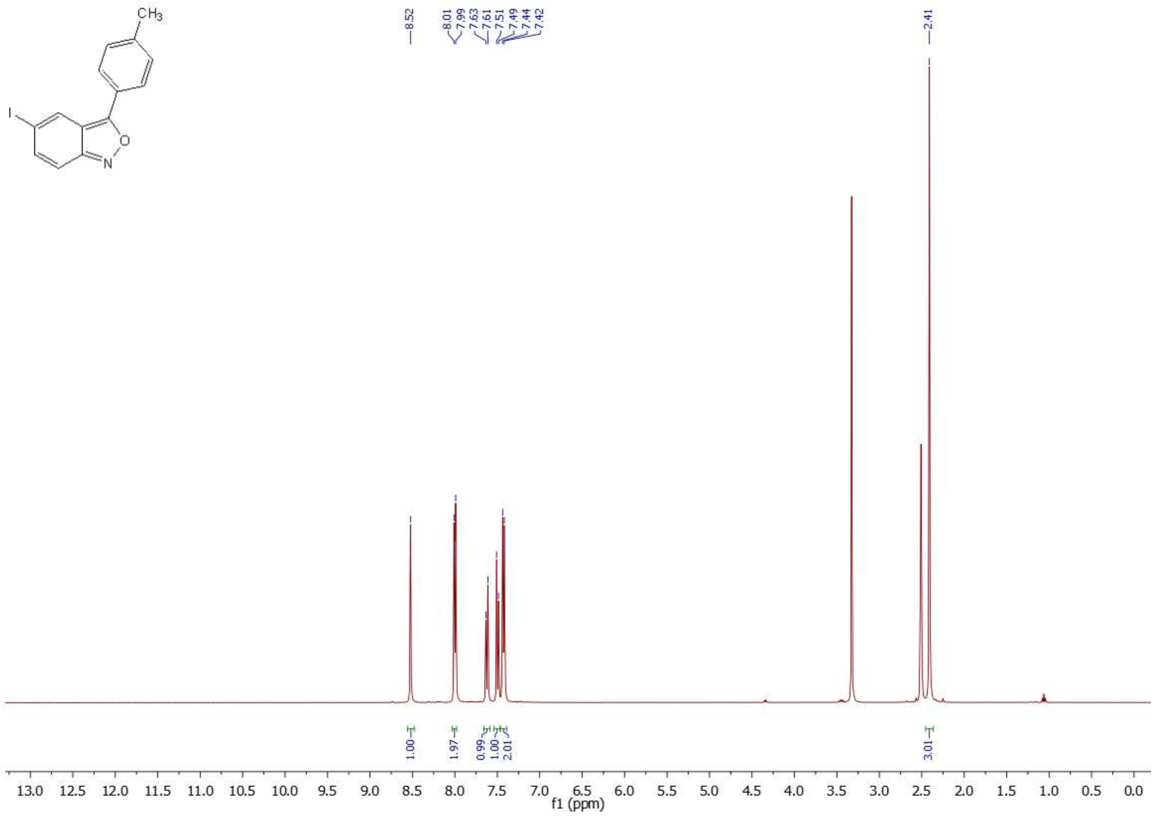

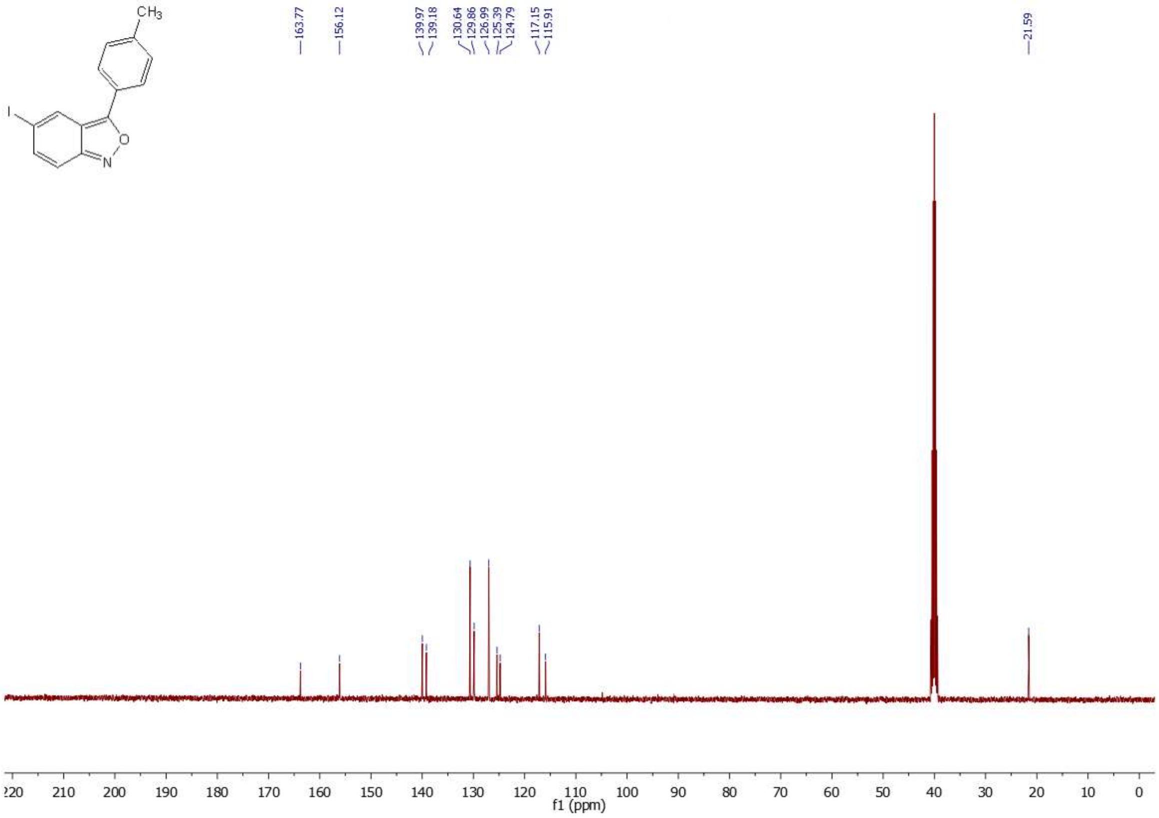

^{1}H NMR spectra of 5-chloro-3-phenylbenzo[c]isoxazole **(3j)**

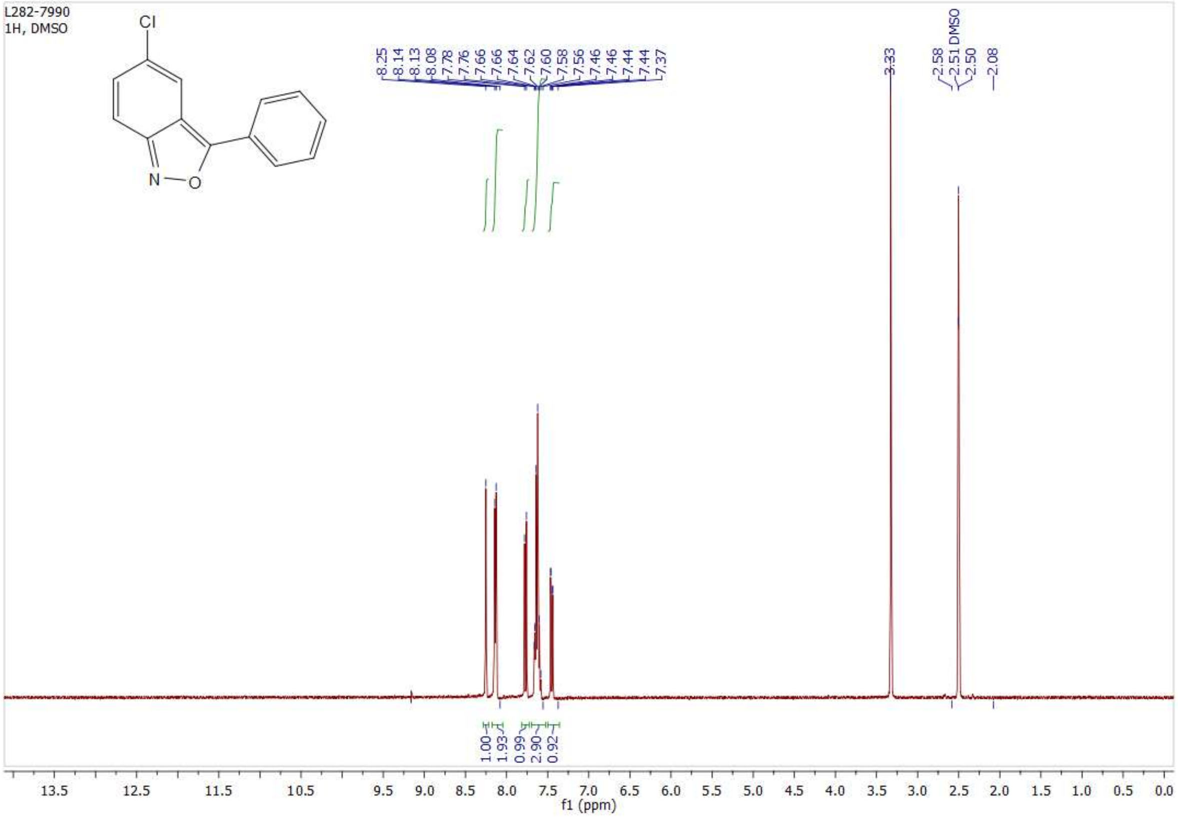

^{1}H NMR spectra of 5,6-dichloro-3-phenylbenzo[c]isoxazole **(3k)**

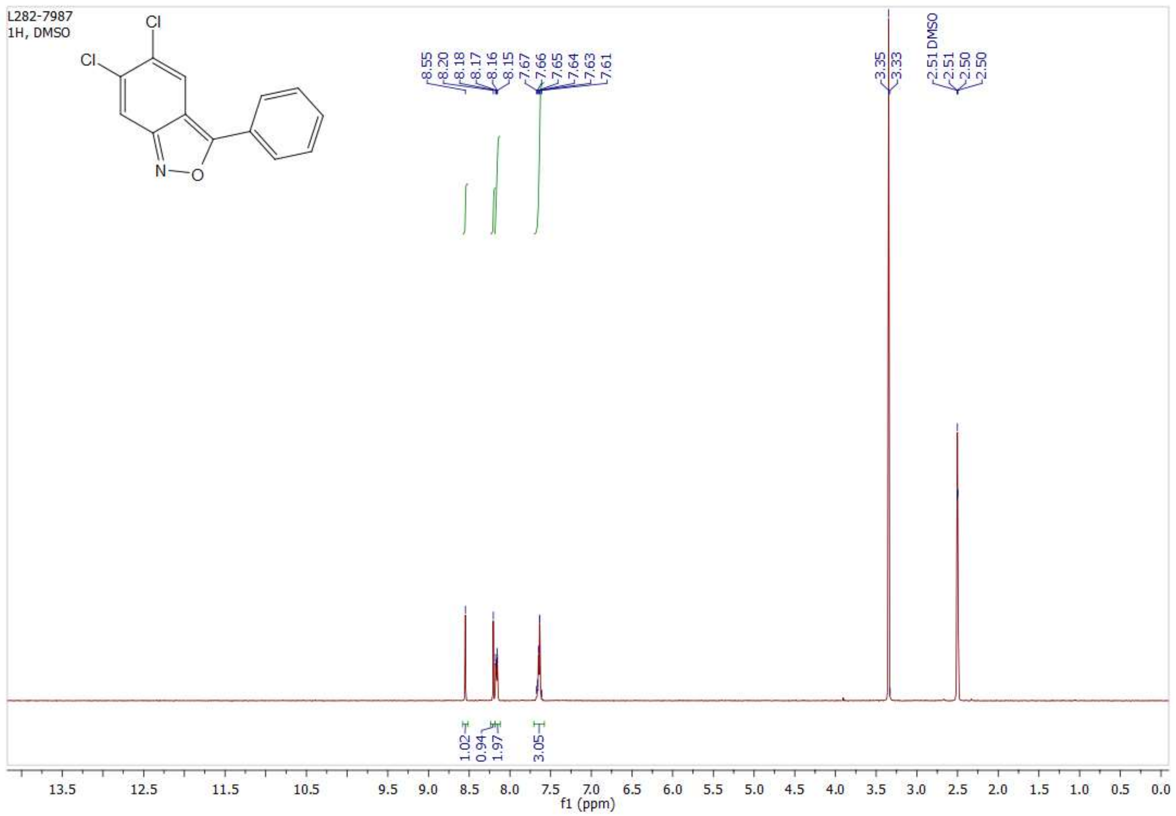

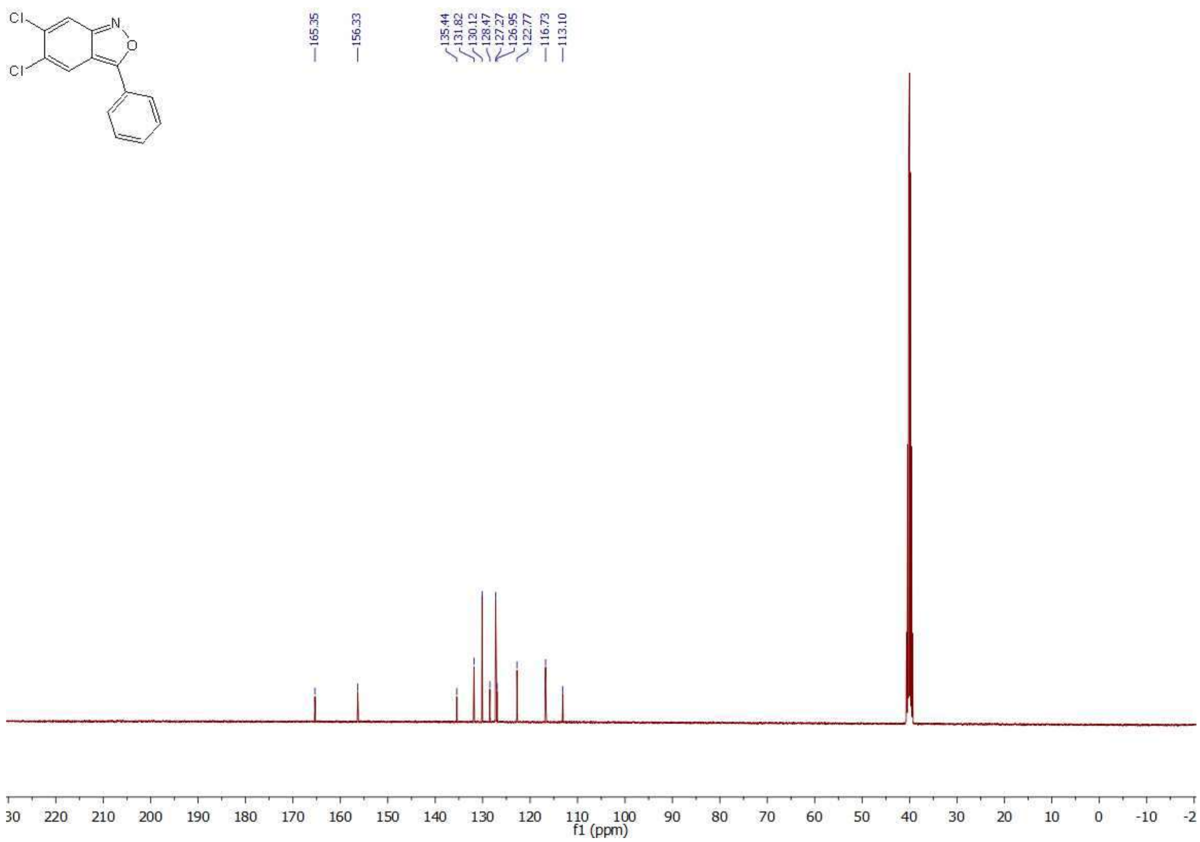

^{1}H NMR spectra of 5-(1,3-dioxolan-2-yl)-3-phenylbenzo[c]isoxazole **(3l)**

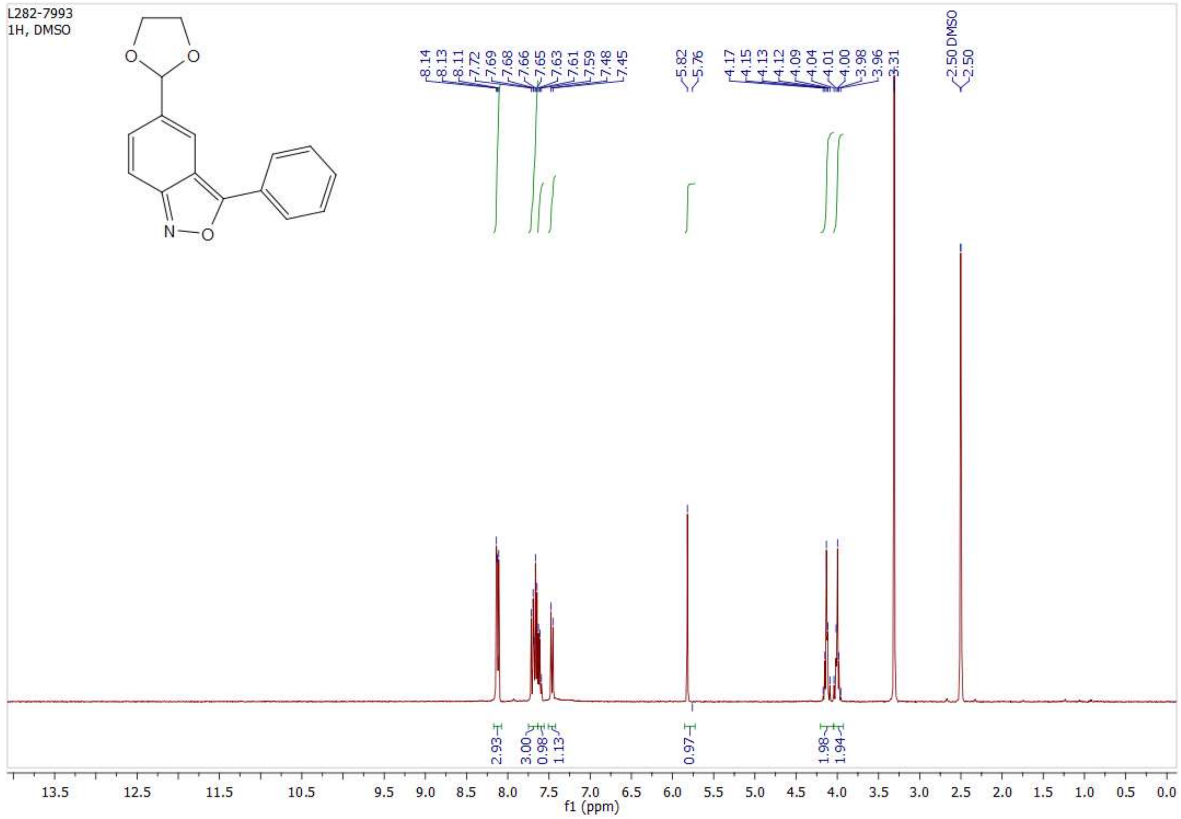

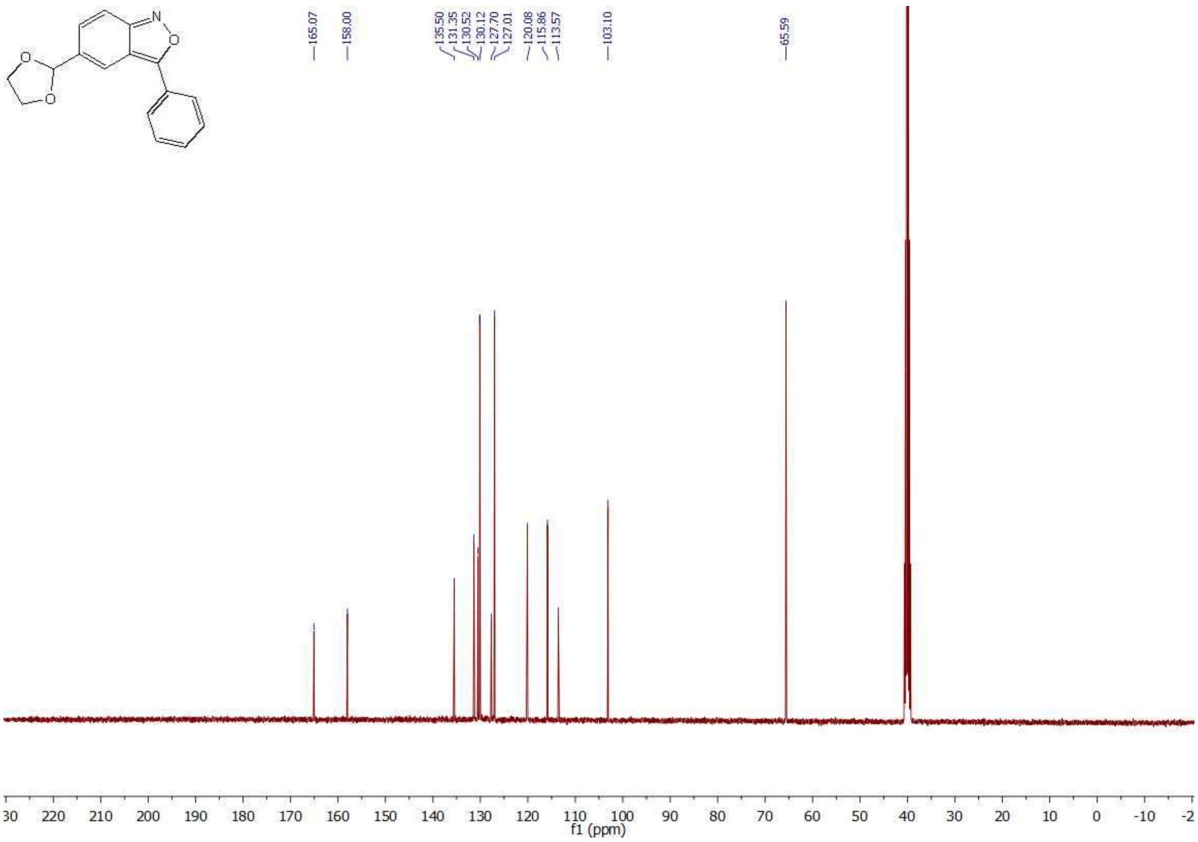

^{1}H NMR spectra of 3-(4-bromophenyl)-5-chlorobenzo[c]isoxazole **(3m)**

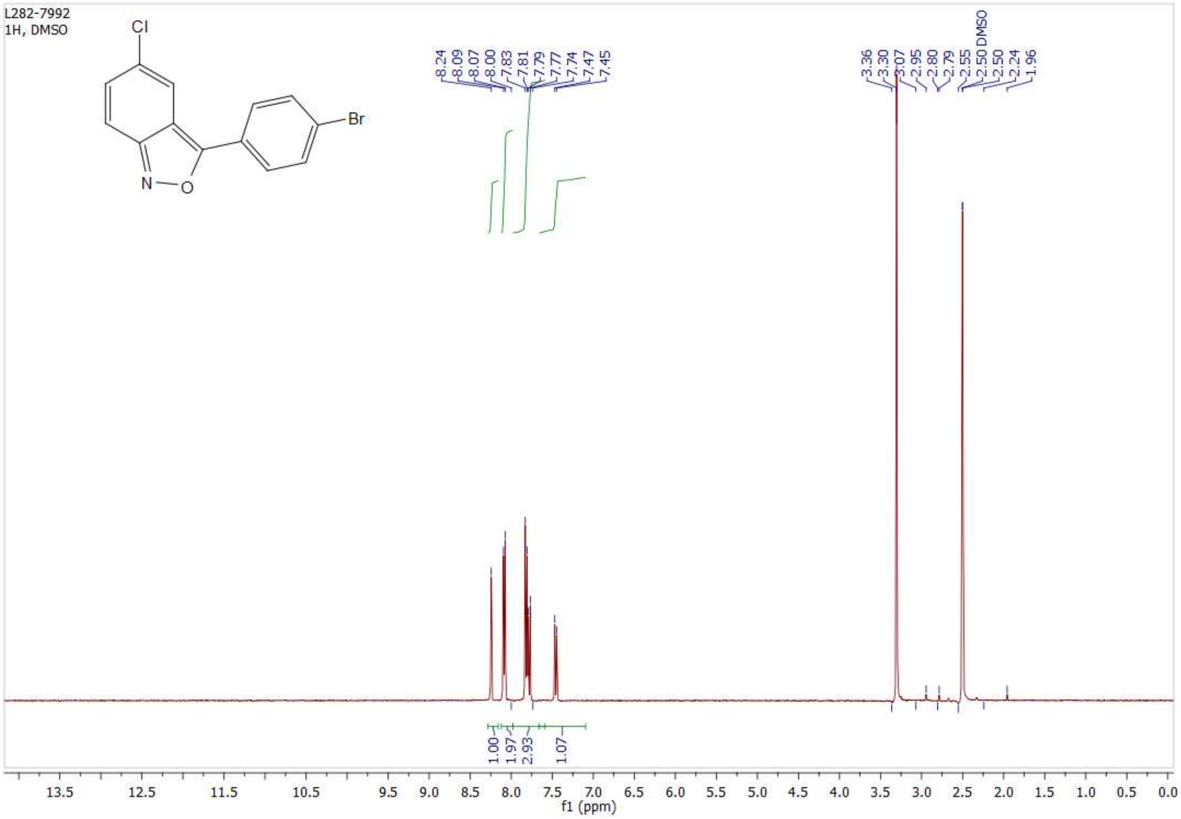

^{1}H NMR spectra of 5-chloro-3-(4-chlorophenyl)benzo[c]isoxazole **(3n)**

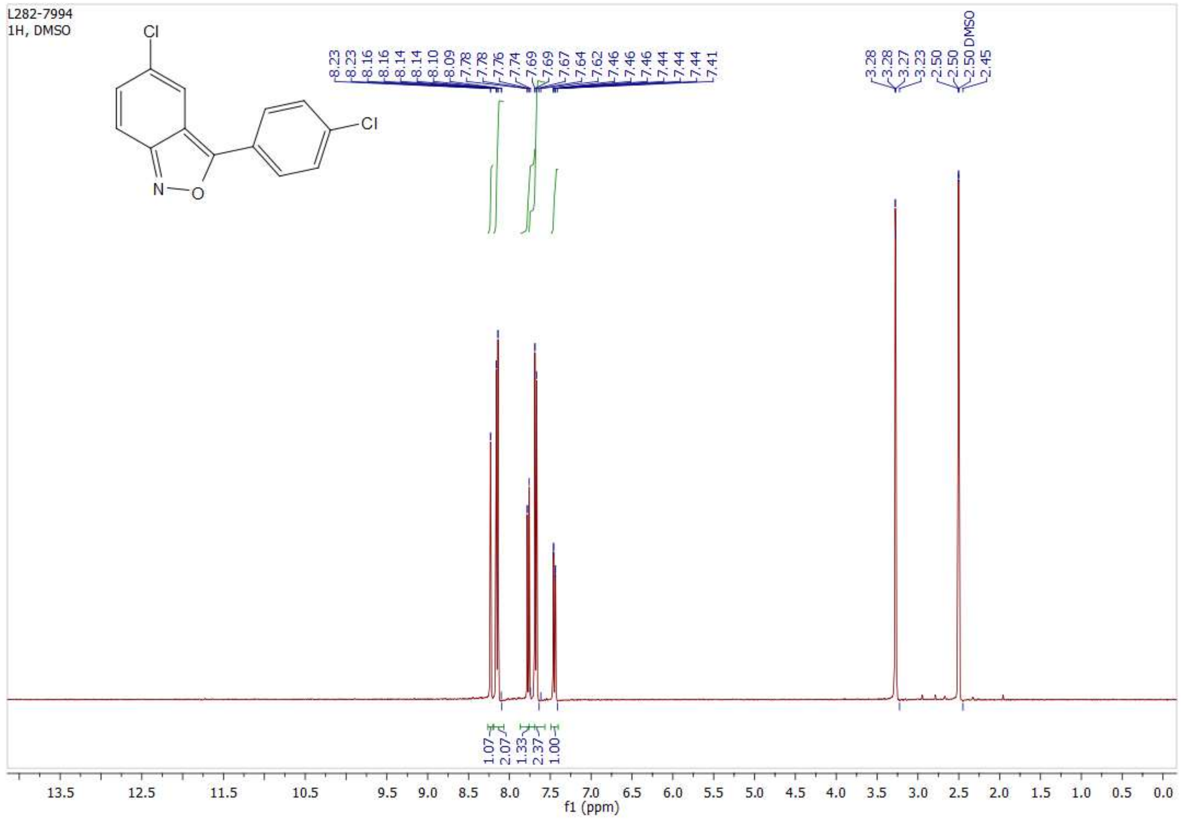

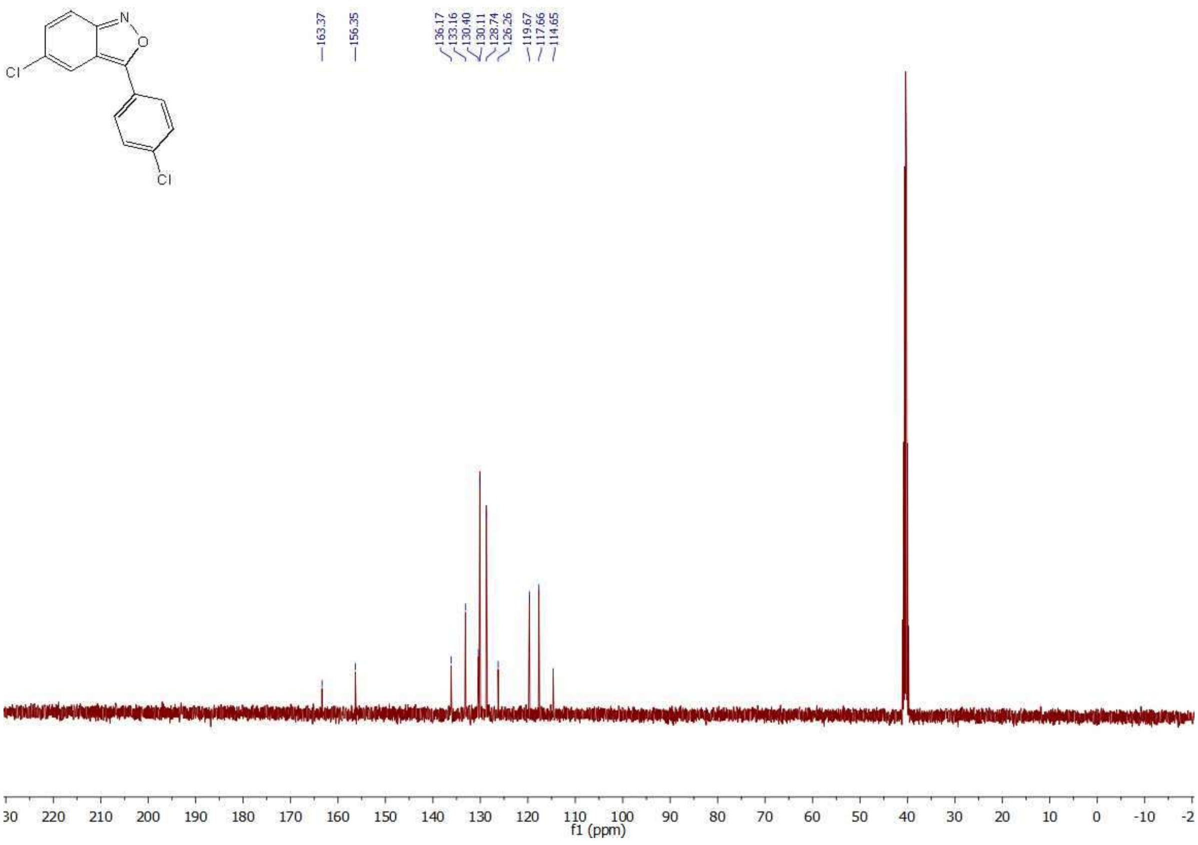

^{1}H NMR spectra of 5-chloro-3-(4-methoxyphenyl)benzo[c]isoxazole **(3o)**

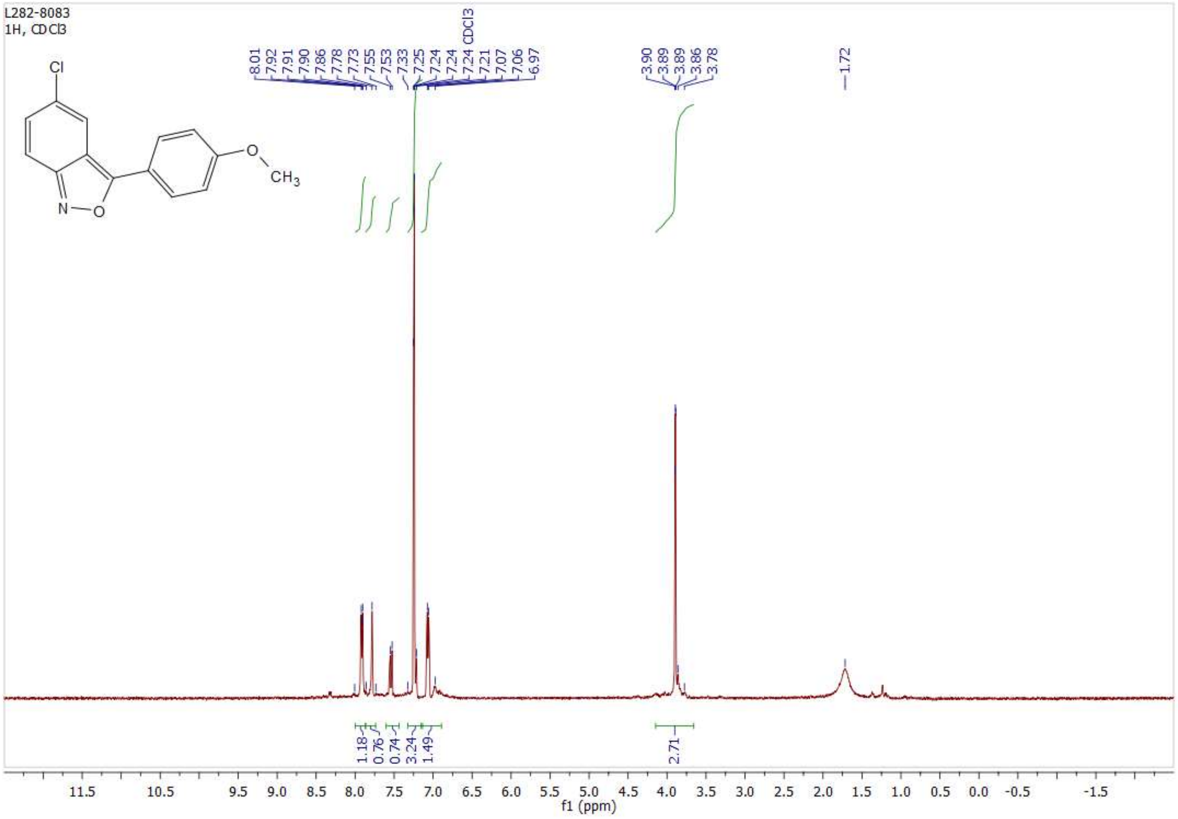

^{1}H NMR spectra of 5,5'-oxybis(3-phenylbenzo[c]isoxazole) **(5)**

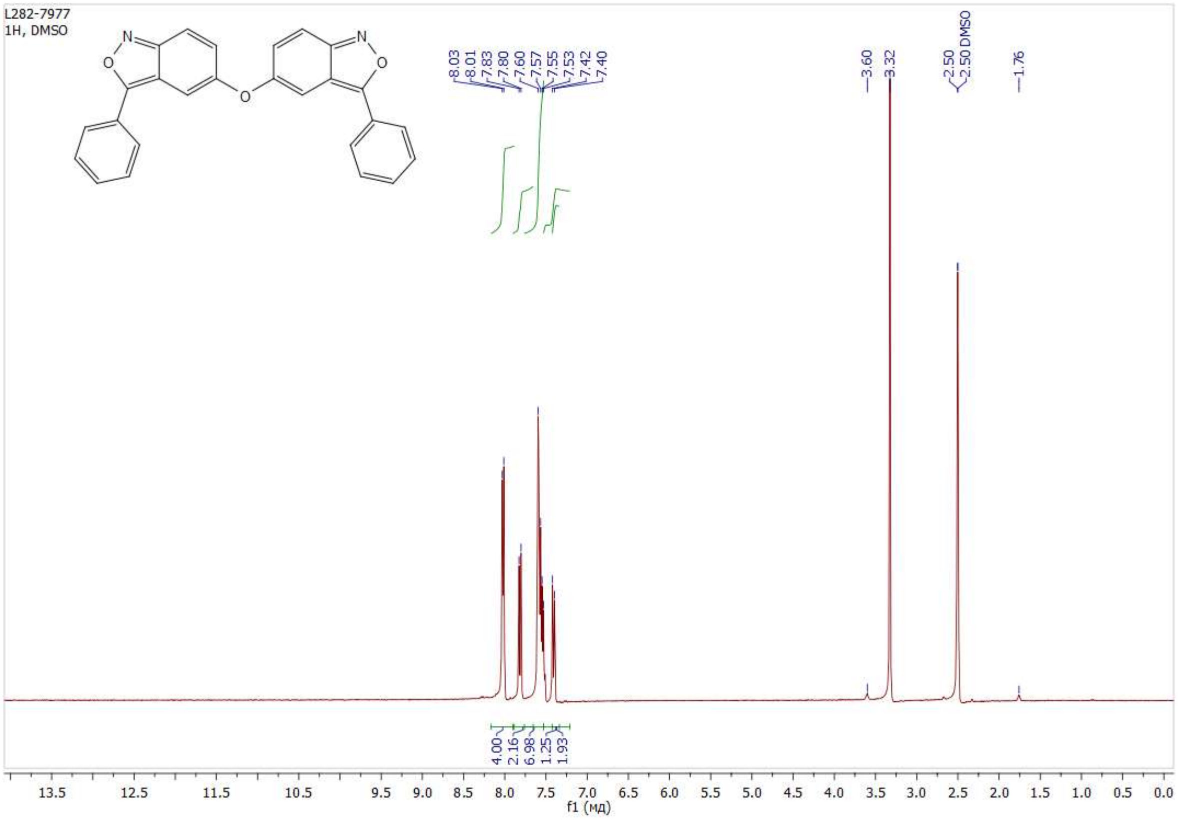

^{1}H and ^{13}C NMR spectra of 4-(5,7-dichlorobenzo[c]isoxazol-3-yl)benzonitrile **(7a)**

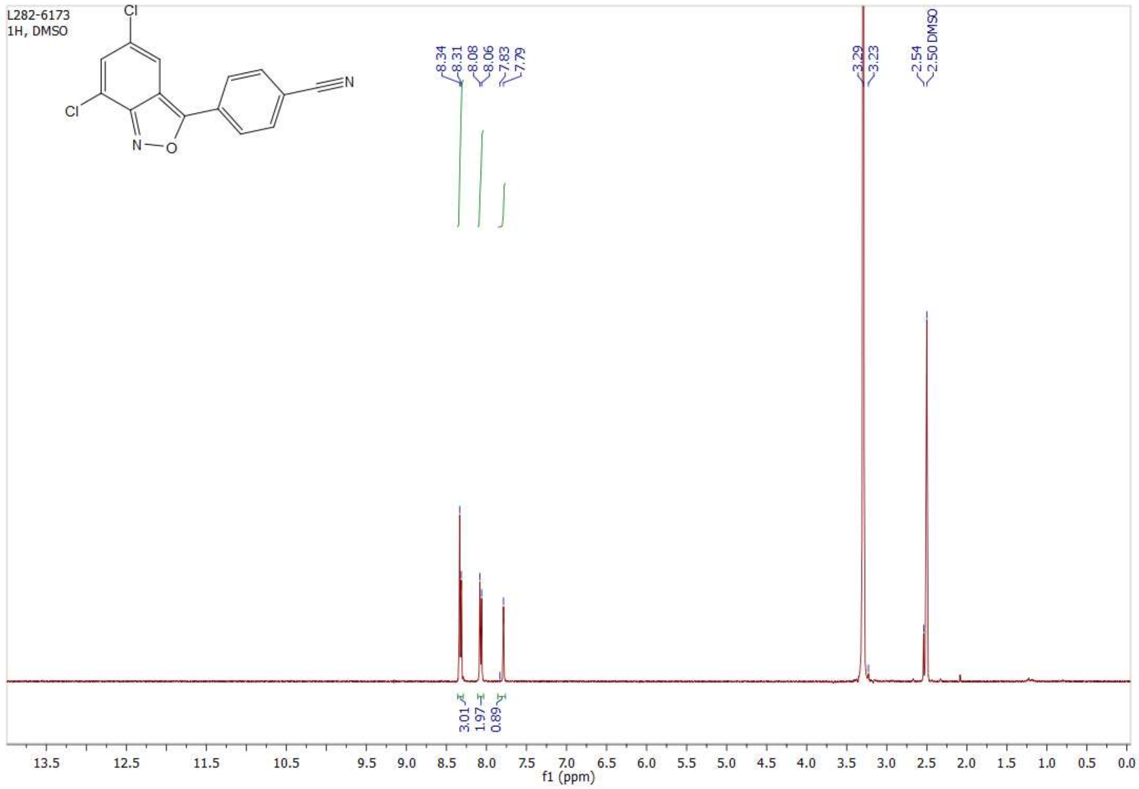

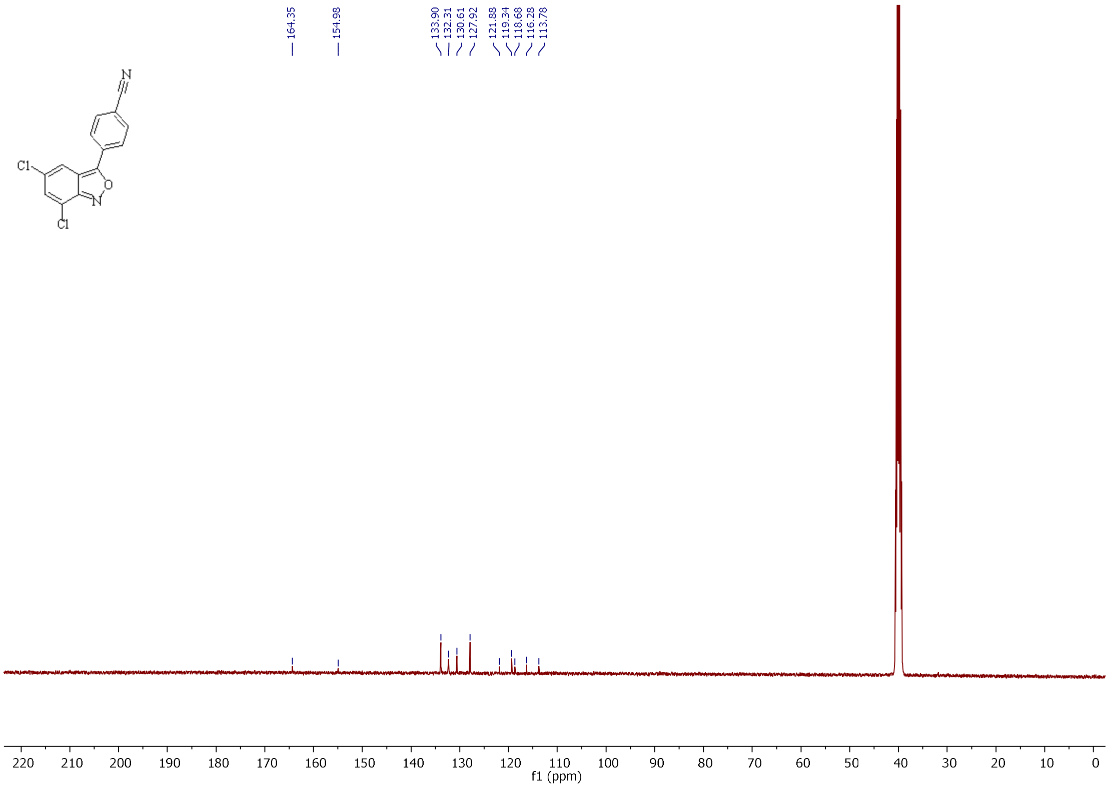

^{1}H and ^{13}C NMR spectra of 4-(5-chlorobenzo[c]isoxazol-3-yl)benzonitrile **(7b)**

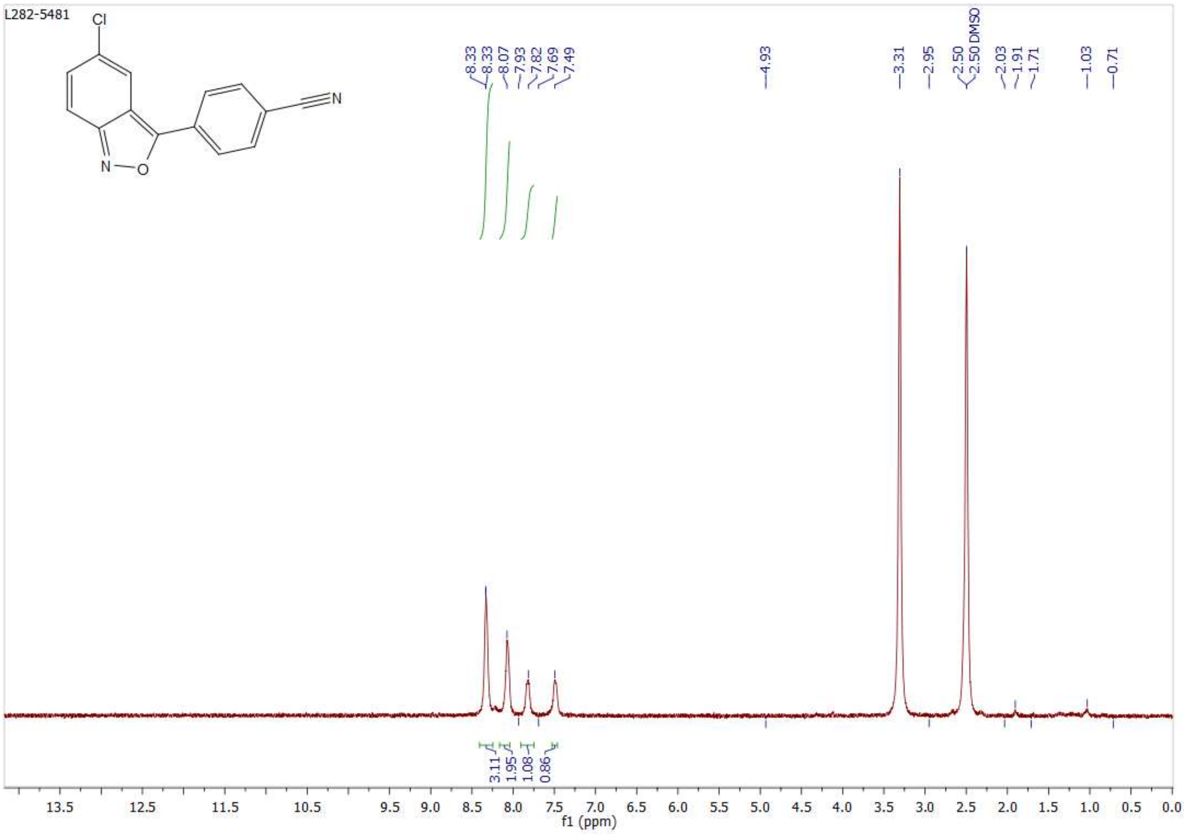

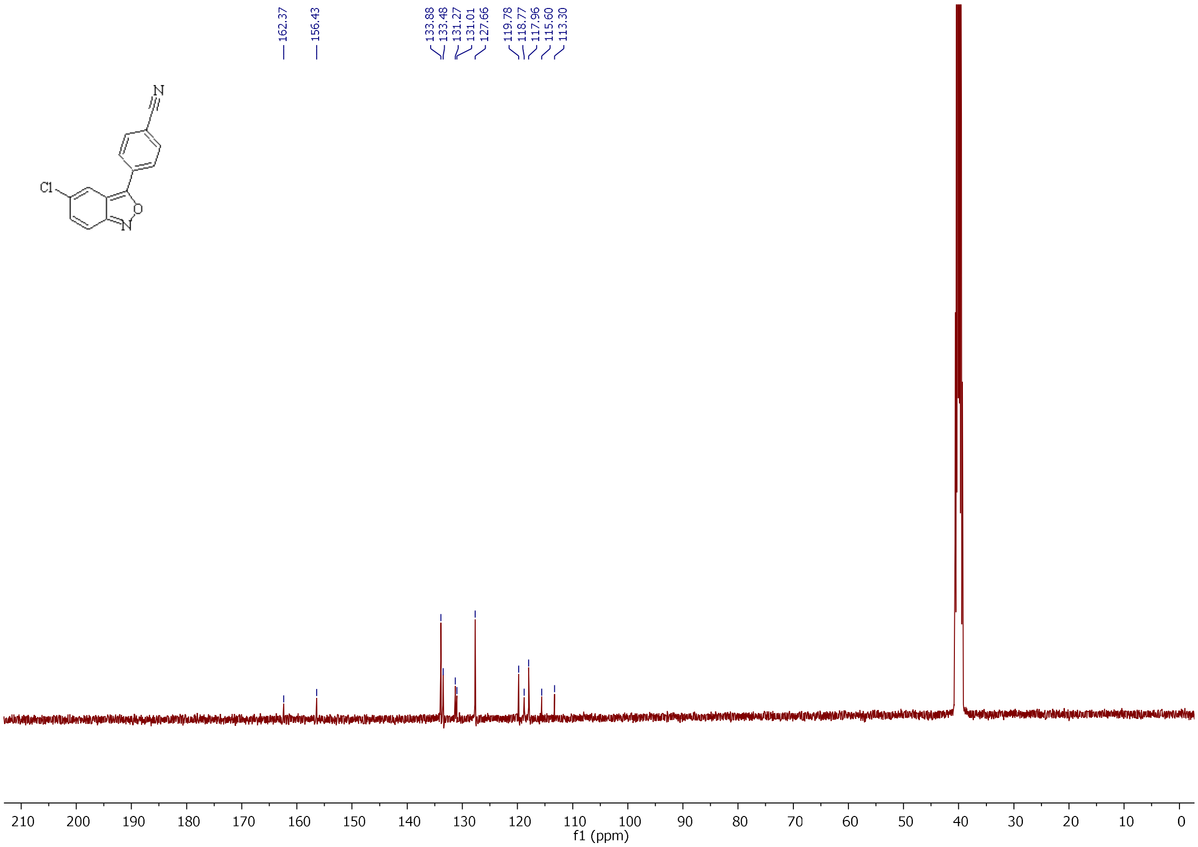

^{1}H and ^{13}C NMR spectra of 4-(5-chloro-6-(5-methyl-1,2,4-oxadiazol-3-yl)benzo[c]isoxazol-3-yl)benzonitrile **(7c)**

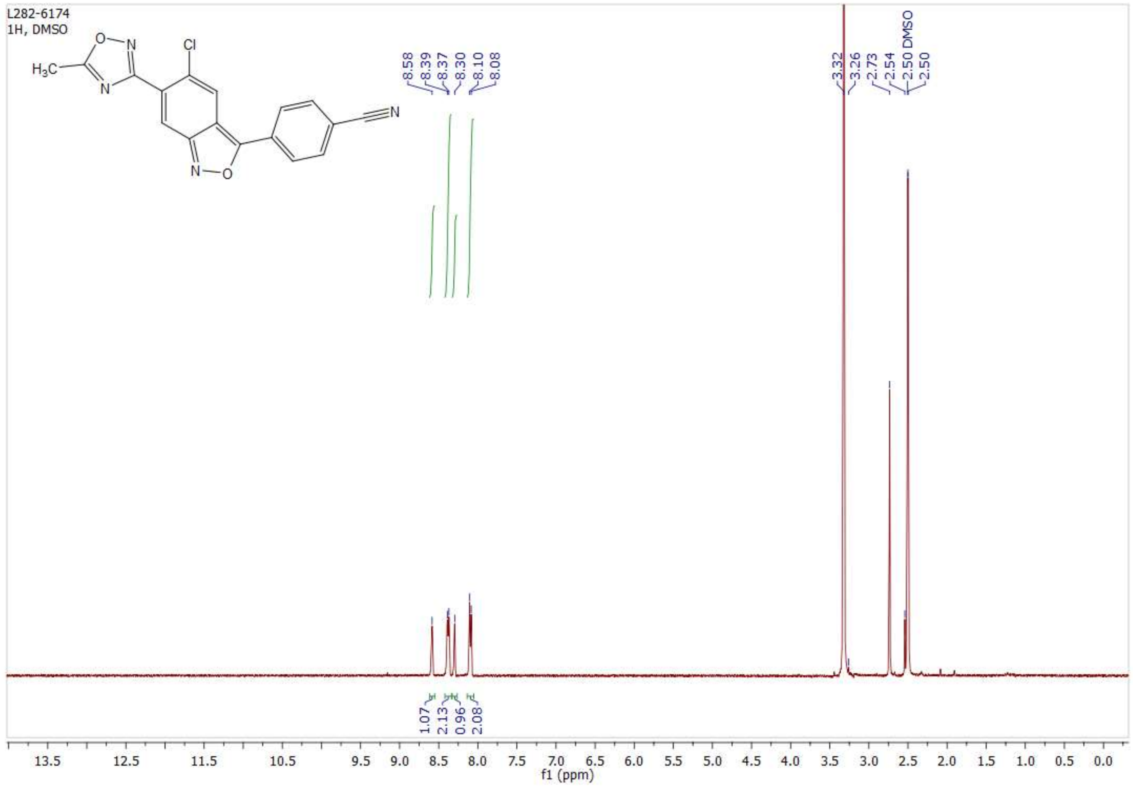

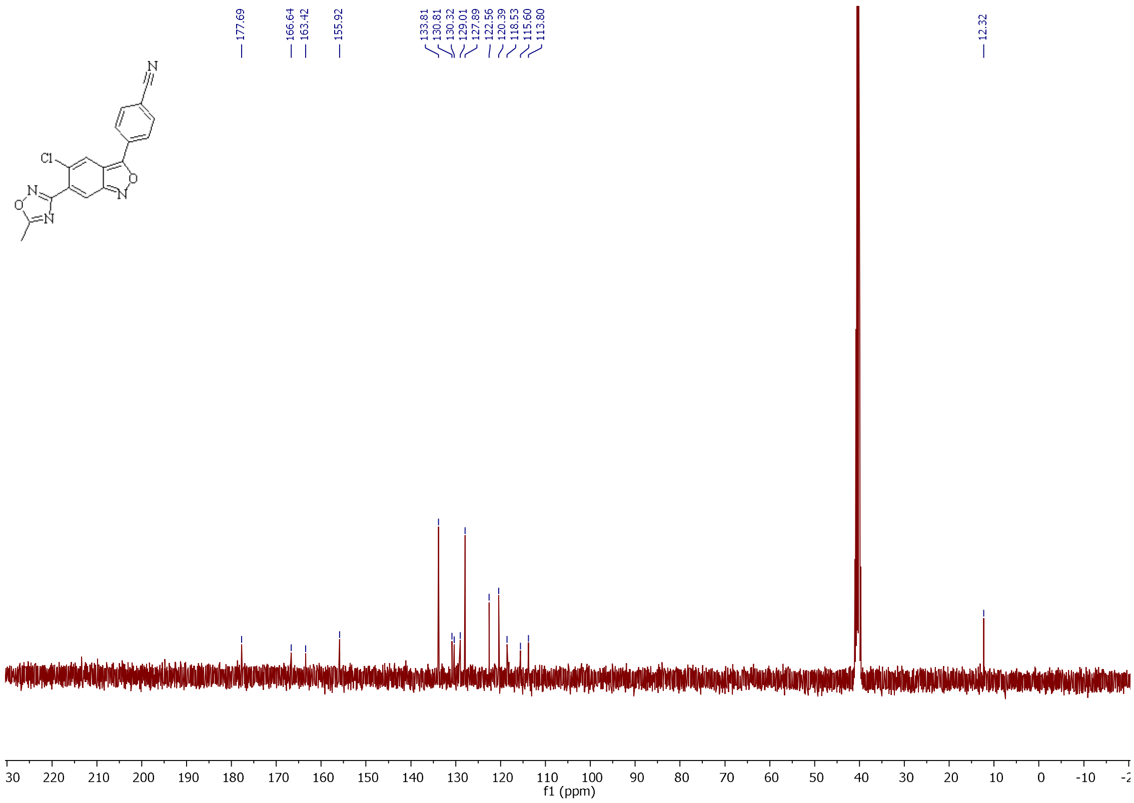

Supplement: Supplementary file 1 — Supplementary file1 (PDF 1805 KB) 1H NMR and 13C NMR spectra for the synthesised compounds and crystallographic data for 3c. [file 11030_2023_10628_MOESM1_ESM.pdf]
